# Reverse engineering environmental metatranscriptomes clarifies best practices for eukaryotic assembly

**DOI:** 10.1186/s12859-022-05121-y

**Published:** 2023-03-03

**Authors:** Arianna I. Krinos, Natalie R. Cohen, Michael J. Follows, Harriet Alexander

**Affiliations:** 1 MIT-WHOI Joint Program in Oceanography and Applied Ocean Science and Engineering, Cambridge and Woods Hole, MA USA; 2 Department of Biology, Woods Hole Oceanographic Institution, Woods Hole, MA USA; 3 Department of Earth, Atmospheric, and Planetary Science, Massachusetts Institute of Technology, Cambridge, MA USA; 4 Skidaway Institute of Oceanography, University of Georgia, Savannah, GA USA

**Keywords:** Pipeline, Protist, Metatranscriptomics, Ecology, Ocean, Marine microbiology

## Abstract

**Background:**

Diverse communities of microbial eukaryotes in the global ocean provide a variety of essential ecosystem services, from primary production and carbon flow through trophic transfer to cooperation via symbioses. Increasingly, these communities are being understood through the lens of omics tools, which enable high-throughput processing of diverse communities. Metatranscriptomics offers an understanding of near real-time gene expression in microbial eukaryotic communities, providing a window into community metabolic activity.

**Results:**

Here we present a workflow for eukaryotic metatranscriptome assembly, and validate the ability of the pipeline to recapitulate real and manufactured eukaryotic community-level expression data. We also include an open-source tool for simulating environmental metatranscriptomes for testing and validation purposes. We reanalyze previously published metatranscriptomic datasets using our metatranscriptome analysis approach.

**Conclusion:**

We determined that a multi-assembler approach improves eukaryotic metatranscriptome assembly based on recapitulated taxonomic and functional annotations from an in-silico mock community. The systematic validation of metatranscriptome assembly and annotation methods provided here is a necessary step to assess the fidelity of our community composition measurements and functional content assignments from eukaryotic metatranscriptomes.

**Supplementary Information:**

The online version contains supplementary material available at 10.1186/s12859-022-05121-y.

## Background

Eukaryotic microbes play diverse and important roles in global ecosystems [1], including grazing processes, primary production, and acting as hosts for diverse and essential symbionts [2]. In ocean ecosystems in particular, the literature on the role of eukaryotic microbes in ecosystem processes continues to expand [3]. This literature provides further evidence that eukaryotic microbes are as important as prokaryotic counterparts when it comes to nutrient cycling and their outsized influence on food webs and community ecology [4–7], and necessitates renewed efforts of understanding the underlying mechanisms.

The ecological relevance of eukaryotic microbes requires careful study of their ecology and distribution, but this can be difficult to execute, both in situ and in the laboratory. The diversity of natural eukaryotic assemblages makes comprehensive surveys difficult to perform in a taxon-specific manner. Taxonomic diversity may be catalogued in the field using 18S rRNA gene amplicons or cell count data, although this neglects functional diversity, which may be leveraged to inform broader understanding of their biogeochemical and ecological roles. Complicating efforts, many eukaryotic microbes are not easily cultivatable in the lab [1, 8], and relying solely on the subset of eukaryotic microbes that we can grow in the laboratory may apply a biased filter to our understanding of these organisms [9]. For these reasons, the use of culture-independent environmental metatranscriptomic and metagenomic sequencing techniques has become a popular and successful method for uncovering new taxonomic and functional diversity in populations of eukaryotic microbes in diverse environments in the field [8, 10, 11].

Metatranscriptomics has become a widespread and promising approach to answer questions about microbial community activity in the environment without prior knowledge or bias [12], and may be used to identify underlying genetic mechanisms driving global phenomena like ocean biogeochemistry [13–15]. Metatranscriptomes provide an accessible means to look at the full suite of genes being expressed by a group of organisms, which may be partitioned by size, site, or phylogenetic origin [16]. Metatranscriptomes can be paired with metagenomes to provide community-level insight into gene expression, and may represent a functional complement to the increasing amount of community composition and novel binned genome data that is available for microbial eukaryotes [10, 11, 17]. However, despite the potential of this approach, the field is relatively new and standardized practices are immature. The first environmental transcriptome, targeting bacterioplankton, was sequenced in 2005 [18], and marine metatranscriptomes began to appear in the literature around 2008 [16, 19]. Metatranscriptomes offer a snapshot of the whole community at the time of sequencing, but the relative proportion of transcripts and their detectability may not always provide meaningful insights into true biological processes, in particular when sequencing depth is low or references are missing from the database [12]. For this reason, databases must be compiled and new computational approaches must and continue to be developed to process and interpret metatranscriptomic data. The collation of laboratory transcriptomic data to a single location and format by the Marine Microbial Eukaryote Transcriptome Sequencing Project (MMETSP) [3, 20] began as a repository effort and became one of the most important databases enabling the identification of marine microbial eukaryotes from metatranscriptomic sequences (e.g. [21–24]). Substantial discoveries have been made using sequenced metatranscriptomes, including novel explanations for persistent gaps in ecological understanding, such as coexistence within a seemingly narrow niche [23], discovering new genes or putative organisms from previously unknown sequences [19], developing a molecular understanding of the basis of coral disease [25], and decoding the complexities of deep-sea hydrothermal vent microbial communities [26]. Metatranscriptomic data availability, in particular for eukaryotic phytoplankton, has been transformed by the *Tara* Oceans Project, which provided an unprecedented amount of sequence information, enabling us to better interpret the ocean genetic landscape on a global scale [27]. Still, metatranscriptome analysis tends to vary substantially between studies, and interpretation can suffer from biases inherent to the technology.

Reliable, reproducible, and broadly available approaches to metatranscriptome analysis have been lacking, particularly in eukaryotic microbial community assessment. Early transcriptome pipelines were designed in the last decade for conventional, well-studied organisms, such as humans and mice, and their microbial communities (e.g. [28]). These pipelines are unlikely to include user-downloadable software, often are focused on annotation, and do not include a mechanism for de novo assembly and processing [28]. A few years later, the first pipeline for uncharacterized microbial communities emerged, but it was presented as a description of the steps needed for metatranscriptome analysis, rather than as software products available to users [29]. The Simple Annotation of Metatranscriptomes by Sequence Analysis (SAMSA) tool, and its second released version, SAMSA2, are among the most recently updated metatranscriptome analysis tools [30]. While this tool is a complete package that can be downloaded and used by scientists, it focuses on rRNA gene removal steps, and does not include assembly steps [30]. In fields such as microbial oceanography, we often require de novo assembly of transcriptome sequences, as the identity of the organisms in environmental samples is not always known, and even for well-understood organisms, comprehensive references may not be available. To date, metatranscriptome pipelines have either lacked accompanying software products or assembly steps necessarily for de novo environmental analysis. As a consequence, the community remains in need of a reliable metatranscriptome analysis tool that is downloadable, reproducible, and includes de novo transcriptome assembly.

The landscape of de novo transcriptome assembly tools is wide, and often there is disagreement about which tool is best to use for a particular application or the average expression level for a sequenced transcript [31]. The Oyster River Protocol (ORP) software was published in 2018 as an answer to this problem, a tool designed for single transcriptomes and meant to combine assembly tools [32]. Using a collection of transcriptome assemblers, the ORP is designed to overcome the challenge of efficiently collating information from multiple assemblers [32]. Further, it uses a collection of *k*-mer sizes, where a *k*-mer is a *k*-sized portion of the transcript used to split the information up into more digestable pieces, to reduce the likelihood that less abundant transcripts would be favored during assembly due to small *k*-mer size or vice-versa with more abundant transcripts and large *k*-mer size. The ORP, however, is a standalone approach to transcriptome assembly, and does not allow the user to simultaneously process multiple samples, nor does it accommodate metatranscriptomes. The ORP does not integrate obviously with downstream annotation metrics, and rather is an approach to combining transcriptome assemblies built using varying *k*-mer lengths.

More recently, it has been demonstrated that de novo co-assembly using multiple transcriptome assemblers improves the quality of single organism-transcriptome assembly [33]. This was shown using a de novo transcriptome assembly pipeline with non-model organism expression data as input to recapitulate the transcriptome of a single species. A BUSCO (Benchmarking Universal Single-Copy Orthologs; [34]) score quality threshold of 50% recovery was used in order to assess the recovery of single organism transcriptomes [33]. BUSCO is a tool used to determine the proportion of lineage-specific single-copy genes found in a genome (or a transcriptome) in addition to initial statistics based only on sequence content rather than evolutionary lineage [34, 35]. When working with single-organism transcriptomes, metrics like BUSCO scores [34] are appropriate for evaluating the completeness of the sequence library of the organism. This differs from the metatranscriptomic context, wherein BUSCOs from potentially multiple organisms are at play for recovery. The authors of the original co-assembly study [33] note that multiple assemblers used at once for a larger co-assembly contribute to higher quality transcriptomic assemblies of RNAseq data, especially when some subset of the highest-performing assemblers is used [33]. These results may help inform multi-organisms metatranscriptomic community data, but they require a transition from consideration of single-organism BUSCO metrics to identification of key features of multiple organisms present in an environmental community. Specifically, rather than validating a single-organism transcriptome with its BUSCO completeness estimates, it is necessary to identify whether the multiple BUSCO-complete single-organism transcriptomes present in a community-wide sample can be accurately recovered. Identifying the most salient and appropriate metrics for the claim that a single organism has been accurately identified and its functions accurately described from a metatranscriptome poses a significant challenge for the field. This is particularly true for environmental community data in which taxonomic boundaries might not be fully resolved in the first place, and culture representatives may not be available. To further complicate matters, even when assembly products can be shown to be “accurate” relative to commonly used metrics such as contig length, percentage of the raw sequencing reads mapping back to the assembly, and the presence of annotated genes with homology to “core” reference genes, they are not guaranteed to offer the best solution to the assembly problem because of the lack of database representatives [36].

The question that remains from single-organism co-assembly studies is why individual transcriptomic assemblers sometimes produce higher-quality or more complete results, and whether redundancy within each transcriptomic assembly skews quality assessment. In order to answer this question, the assembled content shared in the output of multiple assemblers needs to be compared to the new content offered by combining assembly tools. When standardized approaches to assembly and use of appropriate parameters are used and benchmarked, novel insights about the unifying and diversifying aspects of microbial communities can be established [37, 38]. These are typically centered around one or both of the two essential avenues for annotation of sequence material: the taxonomic identity of the sequences and their functional role in the organism. One previous study that focuses mainly on functional role identification via transcript assembly [39] established a comparative workflow, CoMW, for assessing the success of the recovery of database genes from the human gut microbiome, and compared the effectiveness of the assembly-based process of CoMW to assembly-free methods for metatranscriptomes.

Here, we assess the ability of metatranscriptomic assembly methods, and specifically our co-assembler, multiple-sample co-assembly approach to recover *all* transcripts included from existing single-organism transcriptome assemblies. Rather than testing the recovery of identified database genes, we compare our metatranscriptome assemblies to annotated “designer” metatranscriptomes constructed from diverse transcriptome assemblies from a database created using the MMETSP database [3, 20]. This validation workflow is designed to answer the questions: Do studies that use metatranscriptomics to understand community diversity in eukaryotic microbes found in the environment (a) adequately recapitulate the taxonomic and functional diversity found in those communities? and (b) reproduce consistent *sequences* which could be reliably recovered with repeated sampling and assembly? Specifically, the workflow is intended to explore whether sequences for which we have yet to assign a functional annotation are also recovered after reverse engineering raw reads from previously assembled contigs, or whether some may be artifacts of assembly. In addition, we evaluate whether if some assembly tools outperform others, they more likely to outperform in terms of genes from the assembly which can be annotated, length distribution of contigs, or mapping efficiency in recapitulating the raw reads. Doing so is intended to address whether it is acceptable to forgo one or more of these in favor of lower computational requirements.

The community is in need of a protocol for validating popular metatranscriptomic assessments, and a set of recommendations for how best to manage the challenge of minimizing computational assembly costs while maximizing ecological insight extracted from these powerful data. To address these challenges, we have developed *euk*rhythmic, a pipeline which facilitates metatranscriptome assembly with multiple assembly tools and post-processing for environmental sequence analysis in an all-in-one workflow. Here, we describe the *euk*rhythmic pipeline and validate its performance via the construction of simulated metatranscriptomes using a tool we call jEUKebox, and apply it to the assembly and analysis of published metatranscriptomic datasets and simulated metatranscriptomes. Our benchmarking effort using *euk*rhythmic addresses whether assembling metatranscriptomes from a mixed environmental community is comparable to isolating and sequencing particular species or strains of eukaryotic marine microbes and sequencing their transcriptomes individually.

## Methods

Throughout this paper, we use: “designer metatranscriptomes” to refer to the “gold standard” jEUKebox-simulated metatranscriptomic contigs generated from MMETSP reference transcriptomes with known taxonomic annotations, “simulated raw reads” to refer to simulated raw reads from the gold standard, and “reassembled products” to refer to the combined simulated output of metatranscriptome assembly using the *euk*rhythmic pipeline.

### Eukrhythmic pipeline

#### Data cleaning and trimming

Trimming is performed using Trimmomatic version 0.39, a flexible tool that is specifically suited to paired-end next-generation sequencing data, with user-specifiable parameters [40], with a minimum read length of 50 basepairs, a sliding window of length 4 and quality score 2, and a standard list of Illumina adapters (ILLUMINACLIP:<adapter-list>:2:30:7 LEADING:2 TRAILING:2 SLIDINGWINDOW:4:2 MINLEN:50). Optionally, the user may also choose to filter out spike-in sequences, if they were added during extraction, with bbmap [41].

#### Assembly

**Fig. 1:**
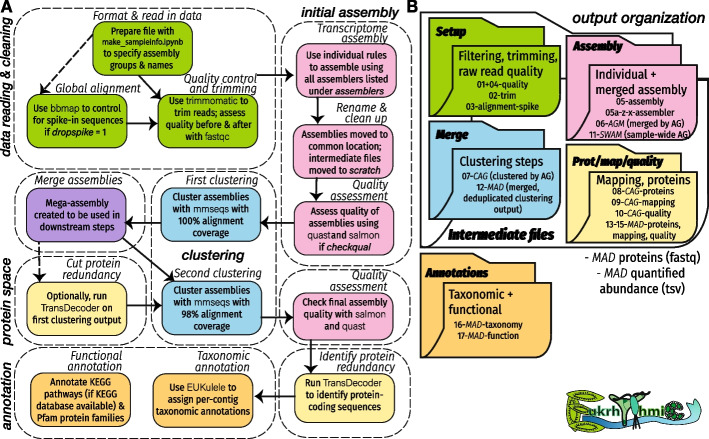
Conceptual diagram of *euk*rhythmic workflow, including **A** major and minor pipeline steps and **B** expected output of the pipeline. Abbreviations: AGM: for each assembly group, assembler products are merged; CAG: AGMs post-clustering; SWAM: sample-wide assembly group merge (all CAGs merged); MAD: merged assembly groups, deduplicated (clustered SWAM)

One major advantage of using the *euk*rhythmic pipeline is the flexibility to use as many (or as few) transcriptome assemblers as is appropriate for the data (Fig. 1). Many different metatranscriptome assemblers are available to researchers and commonly used, and it can be challenging to select the appropriate assembler, given that each often has its own advantages and disadvantages [42, 43]. In *euk*rhythmic, the user may select any combination of assemblers [36, 44–48], and the assembly process is conducted in parallel, as resources allow.

#### Merging and clustering

The consolidation of outputs from the constituent metatranscriptome assemblers is performed in two steps. First, assemblies from the same sample or user-defined “assembly group” (considered a single unit due to some shared characteristic) are concatenated. Inspired by the process adopted by Cerveau et al. (2016) [49], we used the MMSeqs clustering tool [50] to eliminate similar contigs from the combined assembly, first using a sequence similarity threshold of 100% for the shorter sequence in a local alignment to remove identical contigs recovered by multiple assemblers. Next, the pipeline branches into two output types. For the first output type, individual samples/assembly groups (“CAG”, or “clustered by assembly group”), which then undergo a second round of MMSeqs clustering to remove similar contigs at a 98% similarity threshold (defined the same way as above), accounting for potential sequencing errors [49]. Additionally, samples already merged from the assembly process are then merged between samples, such that one combined assembly is produced with all available data, labeled “multiple assembly consolidation” or abbreviated to “MAD” (“multi-assembler deduplicated assemblies”) in the text. We then cluster the combined assembly at the 98% level of similarity using MMSeqs2 as previously described.

#### Protein translation

To accommodate protein-space downstream analysis, such as protein families database (Pfam) annotation [51], protein translation with TransDecoder [52] is supported as part of *euk*rhythmic. Both the output individual sample/assembly group files from the two clustering steps and the single combined assembly are translated to protein sequences.

#### Annotation

While *euk*rhythmic is primarily designed for assembly, the user may optionally elect to annotate the assembly output as part of the pipeline. Presently, the pipeline provides annotation tools including phylogenetic assessment using EUKulele [21], and basic *functional* assessment using the companion tool eggNOG-mapper [53]. To characterize KEGG annotations [54], we grouped results by Kegg Orthology ID (KO). When multiple relevant annotations were associated with a single hit, we assigned counts evenly to the assigned annotations.

### Design of simulated community schema

#### Communities

**Fig. 2:**
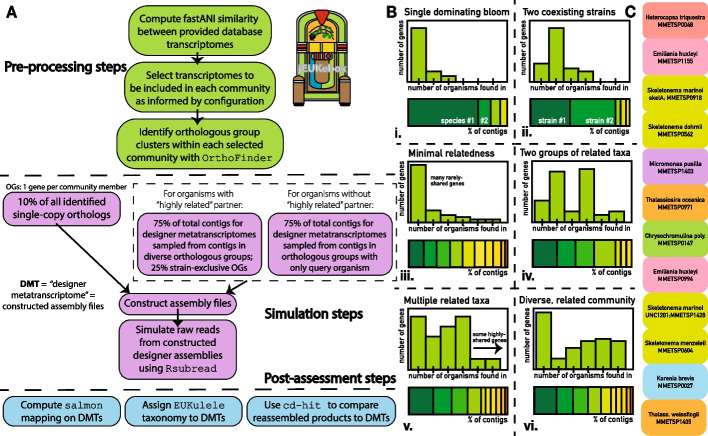
**A** A schematic of the jEUKebox workflow for simulating environmental metatranscriptomes from individual transcriptomes. Laboratory transcriptomes may be curated to fit the individual project; here transcriptomes from the MMETSP dataset were used to simulate reads for the benchmarking of *euk*rhythmic. **B** Conceptual representation of the six targeted community composition simulations (taxonomic representatives are MMETSP Group A in Table 1)

The six simulated communities were designed to have differing complexity and to represent community ecotypes that might be encountered in real-world metatranscriptomic studies. These configurations are summarized visually in Fig. 2 and in terms of their complexity in Table 1 and their taxonomic composition in Table 2. Community 1 was designed to resemble a community dominated by a single organism, thus has the lowest Shannon diversity index and species richness (see calculations in Section “Metrics for assessing community complexity”). Community 2 has a similar species richness value to Community 1 and only marginally higher diversity, since two strains of the same species make up the majority of the sample. Community 3 has the highest number of genes which are not shared between any of the organisms in the sample, but lower diversity than Communities 4 and 6, which have the highest total species diversity. Community 4 has more genes shared between two closely-related groups. Community 5 has the highest total number of reasonably related organisms and shared genes. For MMETSP group B, the list of MMETSP IDs to choose from was selected randomly, and individual community pairings were determined using fastANI similarity (see Section “Simulation of eukaryotic communities using jEUKebox”).

**Table 1:** Calculated diversity metrics for the six simulated MMETSP-based communities used in the analysis

Community	sourmash composite score	Shannon	Richness
1	0.9 ± $10^{-16}$	15.8 ± 0.2	4
2	1.3 ± $10^{-17}$	18.3 ± 0.7	5
3	2.0 ± $10^{-16}$	28.3 ± 0.7	8
4	2.3 ± 0	36.6 ± 0.5	10
5	1.8 ± $10^{-17}$	25.2 ± 0.7	7
6	2.4 ± $10^{-16}$	35.6 ± 0.5	12

The sourmash composite score is an abundance-weighted average of the sourmash distance between two MMETSP transcriptomes. The Shannon diversity index is computed according to [55], and the richness is the number of MMETSP transcriptomes included in the community metatranscriptomes (species richness)

**Table 2:** MMETSP members of each simulated community with the number of orthologous groups which include each organism and the assessed BUSCO completeness of each transcriptome

MMETSP Group	MMETSP ID	Organism	BUSCO Completeness	Number of Orthologous Groups
A	MMETSP0027	*Skeletonema marinoi*	146/255	17057
A	MMETSP0147	*Chrysochromulina polylepis*	119/255	8185
A	MMETSP0448	*Heterocapsa triquestra*	124/255	14377
A	MMETSP0562	*Skeletonema dohrnii*	134/255	10279
A	MMETSP0604	*Skeletonema menzelii*	161/255	9599
A	MMETSP0918	*Skeletonema marinoi*	135/255	9871
A	MMETSP0971	*Thalassiosira oceanica*	152/255	11115
A	MMETSP0994	*Emiliania huxleyi*	113/255	12435
A	MMETSP0995	*Emiliania huxleyi*	99/255	12415
A	MMETSP1403	*Micromonas pusilla*	. 137/255	3779
A	MMETSP1405	*Thalassiosira weissflogii*	151/255	8800
A	MMETSP1428	*Skeletonema marinoi*	152/255	10152
B	MMETSP0321	*Leptocylindrus danicus*	169/255	8235
B	MMETSP0369	*Scrippsiella hangoei-like*	155/255	20932
B	MMETSP0397	*Cyclophora tenuis*	83/255	7306
B	MMETSP0469	*Oxyrrhis marina*	160/255	9456
B	MMETSP0800	*Striatella unipunctata*	100/255	8288
B	MMETSP0884	*Pelagococcus subviridis*	112/255	7134
B	MMETSP0896	*Heterosigma akashiwo*	62/255	3988
B	MMETSP0975	*Pelagomonadales sp.*	171/255	9100
B	MMETSP1117	*Symbiodinium sp.*	129/255	14718
B	MMETSP1338	*Pelagodinium sp.*	141/255	17307
B	MMETSP1349	*Aplanochytrium stocchinoi*	75/255	3747
B	MMETSP1411	*Thalassiosira weissflogii*	144/255	6939

BUSCO completeness is a metric for the quality of the transcriptome based on the presence of shared ancestral eukaryotic genes (of a total of 255 evaluated genes). Reported orthologous groups are based on OrthoFinder [59] analysis on all community members for each MMETSP group; the total number of reported orthogroups was 42,093 for MMETSP group A and 44178 for MMETSP group B

#### Metrics for assessing community complexity

The Shannon diversity index of each community was calculated using the following formula [55]:

$$\begin{aligned} \text {Shannon(community)} = \sum _{j=1}^n p_j \ln p_i \end{aligned}$$

where *n* is the total number of “types” of community members, and *p* is their proportion in their community. Total species richness was reported as the total number of types present in the community.

We used sourmash to compute the pairwise similarity of each MMETSP transcriptome within each community [56]. We additionally introduce another diversity metric to account for the potential similarity of the transcriptomes beyond their taxonomic annotations:

$$\begin{aligned} \text {sourmash composite score} = \sum _{i=1}^{n}\sum _{j=1}^n (1-\text {sourmash score}) \min (p_{i}, p_{j}) \end{aligned}$$

In other words, for each pair of transcriptomes in the community, we weight the sourmash similarity score of the pair of transcriptomes by the abundance of the less-abundant transcriptome in the pair. We report the sum of these weighted scores for each community in Table 1.

### Simulation of eukaryotic communities using jEUKebox

#### Selection of transcriptomes

For each set of simulated eukaryotic communities, 12 transcriptomes from the MMETSP [3, 20] were used. These are summarized in Table 2 for the two selected communities. For “community A”, the IDs, but not the contigs selected, were included based on their features, including some MMETSP IDs of the same species and some of closely related strains. For “community B”, the MMETSP transcriptomes were selected randomly by jEUKebox, with the only constraint being the inclusion of some closely-related taxa. For the random selection built into the pipeline, the only requirement is that some subset of the organisms that went into the communities had to have a highly similar partner in the same community per computed nucleotide similarity score.

#### Similarity computation using fastANI

In order to select “closely-related” transcriptomes for the community specifications, we used fastANI [57] to calculate the average nucleotide-based sequence identity between transcriptomes and identify similar transcriptomes on the basis of having $\ge 80$% average nucleotide identity. Hence, for e.g. community 2 (see Fig. 2), two MMETSP transcriptomes with fastANI similarity $\ge 80$% would be selected.

#### Identifying putative evolutionary relationships with OrthoFinder

In order to test metatranscriptome assembly quality with respect to recovering genes with shared evolutionary origin, but different current annotated taxonomic identity, we used the tool OrthoFinder to identify orthologous groups between the MMETSP transcriptomes, and to include genes from both highly-conserved and relatively rare gene groups in the designer metatranscriptome [58, 59]. As summarized in Fig. 2, the jEUKebox pipeline automates this process by including 10% of all of the identified single-copy orthologs reported by OrthoFinder (orthologous groups with a single gene representative from every transcriptome in the community). Genes are then selected for each one of the organisms in the community according to the following procedure. For genes that *do* have a “highly related” partner with respect to computed similarity (see Section “Similarity computation using fastANI”; fastANI score $\ge 80$%), 75% of the contigs to be included in the designer transcriptome (as prescribed by the desired ratio of the candidate organism in the final metatranscriptomes) were taken from orthologous groups which included more than just the candidate. The remaining 25% were randomly selected from orthologous groups that only contained the candidate. For genes *without* a highly-related partner, 75% of genes were taken from exclusive orthologous groups containing only the candidate. The remaining 25% were randomly selected from orthologous groups shared with other MMETSP transcriptomes.

#### Simulation of raw reads

After creating the designer metatranscriptomes directly from informed random selection of contigs from the MMETSP transcriptomes, raw reads were simulated using the package Rsubread [60]. We chose a read length of 75 base pairs to enable the simReads function to use its inbuilt set of quality scores to randomly determine a sequencing error for the generated raw reads (via the simulate.sequencing.error parameter). We chose a mean fragment length of 180±40 base pairs and generated a 1 million base pair library for the paired sequencing reads which were simulated using the package for each community and trial.

#### Reassembly with eukrhythmic

The raw reads simulated using the simReads function were provided as input to the *euk*rhythmic pipeline. The pipeline was run with default settings as described in Section “Eukrhythmic pipeline” and listed in the configuration file upon downloading the pipeline. Four assemblers were used: rnaSPAdes [36], MEGAHIT [47], metaSPAdes [48], and Trinity [61]. We chose these four assemblers because they each are either designed specifically for the community-level eukaryotic transcriptome assembly problem, or they have been reported to perform particularly well on particular metatranscriptome assembly metrics [62]. rnaSPAdes is built for RNA assembly and performs well with respect to percentage mapping [36, 62], Trinity performs well with respect to number and length of contigs generated [61, 62], MEGAHIT is exceptionally fast [47], and metaSPAdes is designed for community-level sequencing data assembly [48].

### Assessing reassembly quality

#### Assembly statistics

We used the Salmon mapping tool to both quantify the abundance of each contig with respect to the raw reads, and to assess what proportion of the raw reads were represented in the assembled contigs.

We report descriptive statistics for the contigs assembled as a proxy for the quality of the assembled sequences. These include minimum, maximum, mean, and standard deviation of contig length, as well as the N50 metric. We use the definition of the N50 metric as the minimum length among the set of contigs which together constitute 50% of the total length of all contigs in the assembly, as reported by QUAST [63].

#### Clustering reassembled metatranscriptomic proteins with MMETSP-derived designer metatranscriptomic proteins

To determine whether exact sequence matches were shared between the predicted proteins from the metatranscriptome assembly and the proteins from the MMETSP used to create the designer metatranscriptome, we performed mmseqs2 clustering between the two protein sets [50]. We chose the LINCLUST algorithm as implemented in mmseqs2 due to its exceeding low false discovery rate in clustering [64, 65]. In accordance with what was used by authors of mmseqs2, we report these results using minimum coverage of the target sequence (–cov-mode 1) of 90% and a minimum sequence identity of 90%, at which threshold fewer clusters are produced but there is very little chance of a false negative, i.e. two 90%-similar sequences in the dataset which mmseqs2 fails to report.

When evaluating the likelihood of contigs assembled using *euk*rhythmic to cluster with the designer contigs, we based the comparison on the protein predictions from TransDecoder [52] as clustered through mmseqs2. For each full nucleotide contig, we considered it to have “clustered with the designer metatranscriptome” if at least one ORF from TransDecoder was successfully co-clustered with a protein from the designer assembly, even though transcriptome assembly happens in nucleotide space. This enabled us to also quantify what proportion of the contigs from the *euk*rhythmic assembly were not assigned an ORF at all by the TransDecoder software.

#### Assessing metatranscriptomic proteins using BLAST all-by-all comparison

In addition to clustering, we performed an all-by-all BLAST search between the proteins from the original contigs from the MMETSP and the resulting predicted proteins from *euk*rhythmic. An e-value cutoff of $10^{-2}$ was used to catch the top match on the basis of bitscore, and then hits were classified according to their percentage identity and bitscore value.

#### Taxonomic annotations

As performed within the *euk*rhythmic pipeline, we generated taxonomic annotations for both the designer metatranscriptomes and the reassembled products from *euk*rhythmic with the EUKulele tool (version 2.0.3) using the default reference database of contigs from all MMETSP transcriptomes and the MarRef database [3, 20, 21, 66]. We report differences in the number of annotated species and genera from EUKulele in the reassembled products as compared to the sequences which were prescribed to be included in the designer metatranscriptome using the jEUKebox pipeline. We also compare the EUKulele annotations from the designer metatranscriptomes, including false matches on the basis of poor-quality sequences being present in the database and failing to be annotated to begin with, to the annotations of the reassembled products. We perform standard linear regression on the number of annotations for each species, genus, order, and phylum from the designer metatranscriptomes as compared to the reassembled products. We also categorize taxonomic annotations according to whether they were classified correctly, incorrectly (in conflict with the original annotations), or were not classified. We performed a Welch’s 2-sample T-test for independent samples as implemented in scipy [67] to compare the summed abundances of correctly and incorrectly classified and unclassified sequences.

#### Functional annotations

All functional annotations were determined using eggNOG-mapper (version 2.1.3) [53]. Similarly to taxonomic annotations, reported annotations of orthology terms (KOs) from the Kyoto Encyclopedia of Genes and Genomes (KEGG) were compared between the designer metatranscriptomes via annotation of the contigs from the MMETSP and the reassembled products which were retrieved as an output of the *euk*rhythmic pipeline.

Standard linear regression was performed to compare the abundance of KEGG orthology terms in the designer metatranscriptomes as compared to the reassembled products from *euk*rhythmic. The regression and associated probability value was calculated using the implementation in base R [68].

### Assembling and evaluating environmentally relevant metatranscriptomes from the *Tara* Oceans project

We assembled metatranscriptomes from the *Tara* Oceans project [69, 70] as an environmental counterpart to the simulated sequence data. Metatranscriptome samples from three distinct ocean basins were assembled from the highly-diverse small-size fraction surface samples from the *Tara* project: the North Atlantic, Southern Ocean, and Mediterranean Sea; accession numbers are collated in Table 6. We assembled these metatranscriptomes using default parameters to the *euk*rhythmic pipeline and used MEGAHIT and rnaSPAdes, which proved to be the fastest and most accurate assemblers, respectively, in both the present work and other investigations [36, 47]. Three assemblers were selected so as to compare the mutual findings of the three assemblers to the unique sequence content identified by each. We assess the results of the metatranscriptome assembly via percentage mapping via Salmon using the default *k*-mer length of 31, automatic library type selection, and the –validateMappings flag [71], and taxonomic and functional annotations as provided by EUKulele (version 2.0.3) and eggNOG-mapper (version 2.1.3), respectively [21, 53].

These metatranscriptomes were previously analyzed by [22] with transcribed sequences of length $\ge$150 bases assembled using velvet [72] included as part of the “MATOU” database [22]. In order to compare the contigs generated and retained from our multi-assembler approach, we conducted a blastn [73] search with e-value cutoff of 1e-10 to find the top-scoring match of “MATOU” transcribed sequences against our sequences, and compared the contigs that were successfully matched to the database using this method to those that otherwise could be functionally and/or taxonomically annotated. Identified coding sequences of length $>150$ bases were retained for further analysis following [22].

### Reassembling and evaluating previously explored metatranscriptomes from the Narragansett Bay time series

We assembled ten samples from a 2015 metatranscriptomic study from the Narragansett Bay time series [23]. These samples are stored under National Center for Biotechnology Information (NCBI) project accession number SRP055134 and samples were assigned individual accession numbers collated in Table 5. We assembled these metatranscriptomes using default parameters to the *euk*rhythmic pipeline and used MEGAHIT, rnaSPAdes, metaSPAdes, and Trinity [36, 47, 48, 61]. We compared the taxonomic and functional annotations between assemblers to the composition of major taxonomic groups reported by the 2015 study, which used raw read mapping to reference transcriptome assemblies rather than assembling the metatranscriptome itself [23]. We also compare the insights drawn from the simulated metatranscriptomes via jEUKebox to the patterns that emerge from using multiple assemblers on a previously analyzed environmental dataset.

### Data processing and visualization

Output data from the described tools were processed using Python version 3.8.3 [74] and R version 4.1.0 [68]. Figures were generated using plotnine in Python [75] or ggplot2 [76] in R with organization into panels using patchwork 1.1.2 [77]. Statistical analysis on the data was performed with SciPy [67] or with R version 4.1.0 [68].

## Results

Simulated raw reads were created using the jEUKebox pipeline described in the methods (Section “Simulation of eukaryotic communities using jEUKebox”) were processed with *euk*rhythmic. Briefly, reads were trimmed, underwent quality estimation, and were assembled using multiple software tools which were identified or shown in previous studies to perform well with transcribed mRNA sequences, metagenomic data, or both [36, 47, 61], were clustered, and then were functionally and taxonomically annotated with EUKulele (version 2.0.3) and eggNOG-mapper (version 2.1.3) [21, 53]. The full details of the jEUKebox and *euk*rhythmic pipelines are expanded upon in the Materials and Methods (Section “Eukrhythmic pipeline”).

### jEUKebox pipeline generates simulated eukaryotic metatranscriptomes with varying sequence diversity

We developed the jEUKebox pipeline to facilitate the rapid creation of comprehensive mock metatranscriptomic datasets that may be be used to benchmark pipelines and software. Here, we construct marine eukaryotic metatranscriptomes with differing sequence diversity and community complexity leveraging reference data from the MMETSP [3, 20]. We treat the jEUKebox-simulated datasets as a gold standard to assess the performance of the *euk*rhythmic pipelines and the assemblers that it uses. More details about how the pipeline simulates raw reads that resemble the data type generated by marine metatranscriptomic surveys can be found in the Materials and Methods (Section “Simulation of eukaryotic communities using jEUKebox”). We chose two distinct groups of laboratory transcriptomes from the MMETSP [20] for the simulations to ensure that the results were not a product of the specific organisms we selected. For the random selection built into the pipeline, the only requirement is that some subset of the organisms that went into the communities had to have a highly similar partner in the same community per computed nucleotide similarity score (Section “Simulation of eukaryotic communities using jEUKebox”). We also designed the jEUKebox pipeline to include a balanced fraction of common transcripts that had an ortholog expressed by multiple organisms, and we implemented six distinct community configurations so as to simulate a range of species richness and evenness (Fig. 2).

### Eukrhythmic products accurately represent the raw reads

**Fig. 3:**
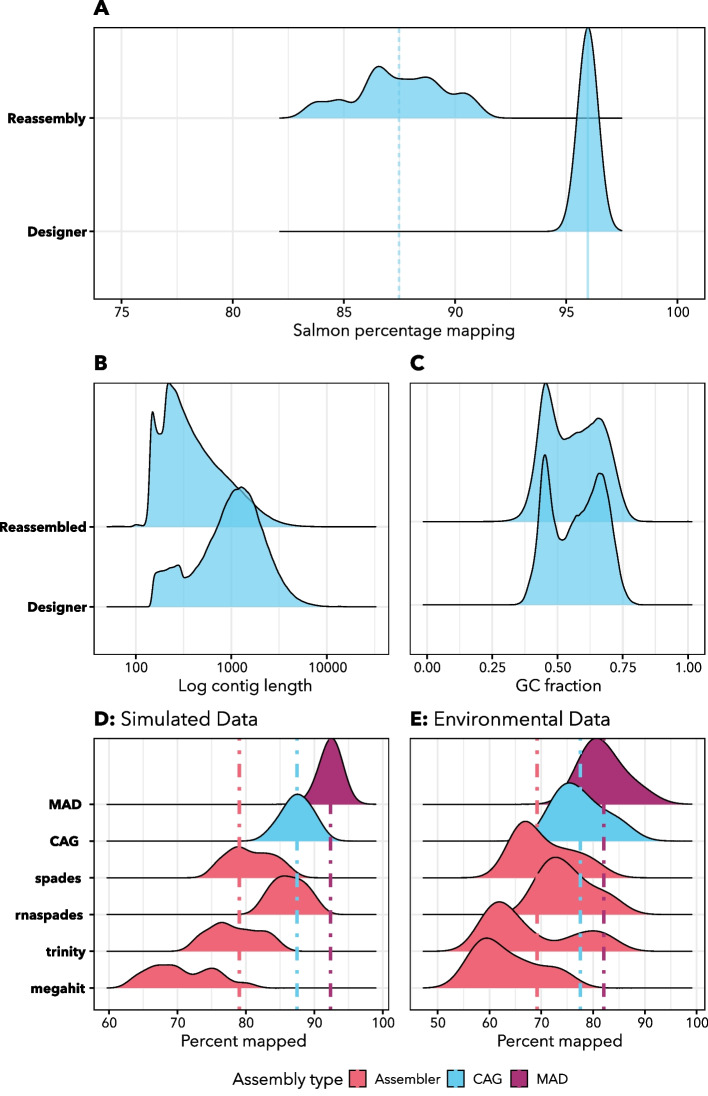
Combined “MAD” assembly improves a suite of assembly statistics relative to individual assemblies. Basic assembly statistics are shown for the *euk*rhythmic reassemblies (per-sample) as compared to the designer metatranscriptomes. **A** Salmon percentage mapping distribution for the designer vs. reassembled metatranscriptomes. **B** Log-normalized contig length distributions compared between designer and reassembled. **C** Per-sequence fraction of GC-content for the designer as compared to the reassemblies. **D** Percentage mapping using Salmon of simulated datasets, separating by the percentage mapping of individual assemblies using each assembler tested (lower distributions), assembly groups clustered into one assembly with multiple underlying assemblers (“CAG”), and all assemblers and assemblies consolidated (“MAD”). All percentage mapping estimates were conducted independently for each set of raw reads from the underlying data. **E** Environmental data from Narragansett Bay using the same comparisons as Panel D. Vertical lines on panels D and E correspond to the mean values of the distribution or set of distributions represented by the color of each vertical line

The *euk*rhythmic pipeline produced reassembled products had similar raw read percentage mapping scores to the designer assemblies. The mapping of simulated raw reads against the *euk*rhythmic reassembled products was lower than against the designer metatranscriptomes against which they were simulated, with $87.5\pm 2.0$% of simulated raw reads mapping against the *euk*rhythmic reassembled products and $96.0\pm 0.2$% against the designer assembly (Fig. 3A-C; Table 3). This discrepancy is likely due to the error introduction step in the raw reads or conflicts between different raw read placements in candidates for reassembled products which could not be resolved by the assembler. These patterns were reproduced in the environmental dataset we tested [23]: both the MAD ($82.1\pm 3.8\%$) assembly and the multi-assembler clustered assembly (“CAG”; $77.6\pm 4.5\%$ mapped) outperformed any individual assembler with respect to percentage mapping (Fig. 3D-E). In our simulated data, rnaSPAdes had the highest average percentage mapping of any assembler, and MEGAHIT had the lowest (Fig. 3D), but patterns were slightly different in the environmental dataset [23]. While MEGAHIT still underperformed relative to the other assemblers with respect to percentage mapping (Fig. 3D, E), comparisons between the remaining assemblers were less straightforward. rnaSPAdes showed the highest individual performance ($75.0\pm 4.7\%$ mapped), followed by SPAdes ($70.5\pm 5.4\%$ percent mapped). However, Trinity performed better in some samples than in others, hence showed higher dispersion in percentage mapping values ($67.8\pm 8.6\%$).

**Table 3:** Resulting assembly size and taxonomic, functional, and core content recovery of jEUKebox outputs after raw read simulation and re-assembly with *euk*rhythmic

Community	Simulated Contigs	Number of Assembled Contig Clusters	Assembly Size (MB)	MMETSP Genera	Designer Genera	Recovered Genera	MMETSP Species	Designer Species	Recovered Species	Designer Distinct KOs	Recovered Distinct KOs
1	51741 $\pm$ 6031	32.6 $\pm$ 3.6	59489 $\pm$ 4825	3.8 $\pm$ 0.5	4.4 $\pm$ 1.1	3.1 $\pm$ 1.1	3.9 $\pm$ 0.4	4.9 $\pm$ 0.6	3.5 $\pm$ 1.3	3435.1 $\pm$ 429.6	1941.2 $\pm$ 161.5
2	44180 $\pm$ 3486	26.1 $\pm$ 2.6	49354 $\pm$ 4948	3.2 $\pm$ 0.9	4.4 $\pm$ 1.2	3.4 $\pm$ 0.7	3.4 $\pm$ 0.7	4.6 $\pm$ 1.1	3.2 $\pm$ 0.7	3422.5 $\pm$ 412.1	1816.9 $\pm$ 188.8
3	47862 $\pm$ 7756	28.0 $\pm$ 5.2	53379 $\pm$ 6921	4.4 $\pm$ 0.9	5.0 $\pm$ 0.0	3.6 $\pm$ 0.9	4.5 $\pm$ 0.9	5.2 $\pm$ 0.5	3.2 $\pm$ 0.7	3346.4 $\pm$ 369.2	1879.2 $\pm$ 154.7
4	49911 $\pm$ 6524	29.5 $\pm$ 4.7	57262 $\pm$ 6752	5.1 $\pm$ 1.4	5.9 $\pm$ 1.1	3.2 $\pm$ 1.0	5.8 $\pm$ 0.9	6.6 $\pm$ 0.9	3.4 $\pm$ 0.9	3284.8 $\pm$ 204.2	1895.8 $\pm$ 149.2
5	44262 $\pm$ 6254	25.9 $\pm$ 3.2	50042 $\pm$ 5395	3.8 $\pm$ 0.9	4.6 $\pm$ 1.1	2.8 $\pm$ 0.7	3.9 $\pm$ 0.8	5.0 $\pm$ 0.9	2.8 $\pm$ 0.7	3226.0 $\pm$ 545.6	1720.6 $\pm$ 127.9
6	52795 $\pm$ 5152	31.2 $\pm$ 2.9	59826 $\pm$ 4076	5.6 $\pm$ 1.3	6.2 $\pm$ 1.2	2.8 $\pm$ 0.7	6.4 $\pm$ 0.9	7.2 $\pm$ 0.7	3.2 $\pm$ 1.0	3049.9 $\pm$ 355.6	1882.6 $\pm$ 156.9

Four assemblers were used in this analysis which were then clustered together using default *euk*rhythmic settings. The mean and standard deviation of four trials of each community and list of MMETSP IDs is presented. We also show the number of genera that were (1) originally included via transcriptomes leveraged from the MMETSP (2) identified using EUKulele within the simulated metatranscriptomes and (3) recovered in the reassembled data after application of the *euk*rhythmic pipeline. For functional KO IDs, only the designer assemblies and the *euk*rhythmic reassembled products could be compared. A version of this table in which the two distinct communities designed from the MMETSP (combining the two contributes to relatively high standard deviation) are presented separately is provided as a supplement

More information: https://github.com/AlexanderLabWHOI/jEUKebox

**Table 4:** Comparison of the average length of sequences in the designer metatranscriptomes as compared to the *euk*rhythmic reassemblies

Assembly	Sequence Type	Average Length	Average Fraction GC Content
Designer Assemblies	Nucleotide	$1278.7\pm 1027.4$	$0.57\pm 0.10$
Reassembled Products	Nucleotide	$539.9 \pm 564.3$	$0.56 \pm 0.10$
Designer Assemblies	Protein	$276.3 \pm 261.7$	-
Reassembled Products	Protein	$165.1 \pm 157.7$	-

Both the average length of nucleotide sequences and protein sequences as predicted by TransDecoder are provided, as well as the average fraction of GC-content for nucleotide sequences

Average contig length tended to be significantly shorter in the *euk*rhythmic reassemblies as compared to the designer metatranscriptomes, although there was considerable variability (Fig. 3). The average length of open reading frames (ORFs) predicted by the TransDecoder tool was also smaller in the *euk*rhythmic reassemblies as compared to the original sequences retrieved from the MMETSP transcriptomes (Table 4; Fig. 3). Although still substantially shorter than the designer metatranscriptomes, sequences in the *euk*rhythmic products that were recovered by more than one assembler according to mmseqs2 clustering had progressively longer length (median length 334 base pairs for clusters represented by a single assembler, median length 960 base pairs for clusters represented by all four assemblers; between-distribution t-test $p<0.001$; Fig. 3). These longer contigs had high fidelity with the raw reads, as evidenced by the agreement of multiple assembly approaches, hence were likely to be longer sequences interrupted by fewer conflict instances.

### Less stringent clustering slightly decreases identified annotations

**Fig. 4:**
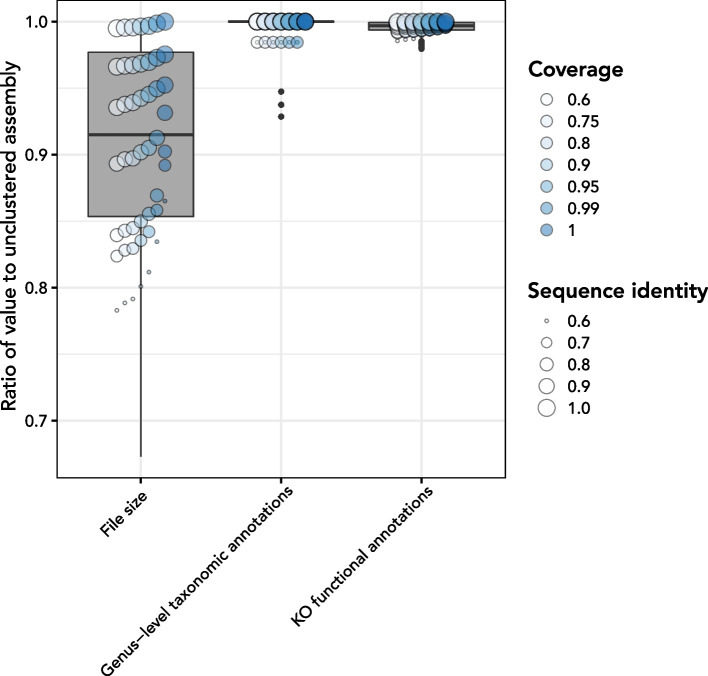
Clustering the designer assembly scales assembly size and number of annotations recovered. Clustering was performed on the original “designer metatranscriptome” set of contigs from the MMETSP references using the mmseqs2 tool [50]. The effect of coverage level (color) and percentage identity (size) via mmseqs2 on the file size, number of sequences annotated at the genus level, and number of sequences with functional annotations relative to the unclustered assembly was examined. The boxplot underlying each set of points highlights the distribution of ratios relative to the unclustered assembly. *euk*rhythmic uses a coverage level of 0.98 and sequence identity of 1 for mmseqs2 clustering. See Additional file 1: Fig. S1 for a more detailed graphical summary of the influence of sequence identity and coverage on the size of the recovered assembly and its functional and taxonomic annotations

*euk*rhythmic reduces the redundancy of the identified contigs for the merged assembly via clustering, hence reducing computational complexity of downstream operations on the smaller multi-assembler, multi-sample assembly file. Applying clustering directly to the designer metatranscriptomes revealed that substantial protein-space clustering only slightly decreases the unique annotations extracted from the dataset. For example, mmseqs2 clustering with a sequence identity threshold of 0.6 and coverage threshold of 0.6 in coverage mode 1 reduced the number of contigs in the assembly by an average of 23.7% and reduced the assembly file size by an average of 21.7%, but only reduced average identified KEGG database functional annotations by 1.4% and did not result in the loss of any species from the dataset via clustering (Fig. 4). By default, *euk*rhythmic uses a conservative approach of 100% sequence identity and 98% coverage for the most lenient clustering step, but we found in this test that values of 80% for both coverage and sequence identity could considerably reduce total file size without considerably changing unique annotations (Additional file 1: Fig. S1). Given this substantial reduction in file size without loss of the majority of annotations, more stringent clustering thresholds may be warranted, especially in datasets with many samples or high sequencing depth.

### Eukaryotic metatranscriptome assembly accurately recapitulates simulated taxonomic diversity

On the whole, all assemblers performed well with respect to recovery of the major taxonomic annotations from the simulated metatranscriptomes. 94.8±2.2% of all recovered contigs were assigned genus-level annotations by the EUKulele tool that matched genera found in the selected MMETSP transcriptomes used to simulate the metatranscriptomes (97.7±2.2% of annotated contigs). In general, the number of annotations in conflict with the genus-level annotation assigned based on the MMETSP was similar in the designer metatranscriptomes as compared to the reassemblies generated by *euk*rhythmic. The computed linear regression between the genus-level annotations from the designer assemblies and the *euk*rhythmic re-assemblies was nearly one-to-one: $\text {Reassembly} = -1353+1.02(\text {Designer})$; $R=0.95$; $p=<8.2e-184$; note that the intercept is relative to total abundances on the order of $10^{5}$. This indicated that the total abundance of each genus-level annotation as assessed by Salmon quantification matched well between the designer metatranscriptomes and the reassembled products from *euk*rhythmic.

**Table 5:** Sample IDs and accession numbers for Narragansett Bay samples. Descriptive information about the sample conditions are reproduced from [23]

Sample ID	Assembly Group	Accession Number	Experimental Context
NarBay_A	NarBay_A	SRR1810207	Nitrate added (+N)
NarBay_B	NarBay_B	SRR1810208	-N
NarBay_C	NarBay_C	SRR1810209	Phosphate added (+P)
NarBay_D	NarBay_D	SRR1810210	-P
NarBay_E	NarBay_E	SRR1810211	No amendment
NarBay_S1	NarBay_S1	SRR1810799	Environmental sample 1
NarBay_S2	NarBay_S2	SRR1810204	Environmental sample 2
NarBay_S3	NarBay_S3	SRR1810801	Environmental sample 3
NarBay_S4	NarBay_S4	SRR1810205	Environmental sample 4
NarBay_S5	NarBay_S5	SRR1810206	Environmental sample 5

**Table 6:** Sample ID and accession numbers for the 15 *Tara* Oceans metatranscriptomes assembled as part of this project, including the ocean basin they were sampled from

Accession Number	Sample ID / Assembly Group	Ocean Basin
ERR1712028	SO_SRF_SMALL_ERR1712028	Southern Ocean
ERR1719157	SO_SRF_SMALL_ERR1719157	Southern Ocean
ERR1740115	SO_SRF_SMALL_ERR1740115	Southern Ocean
ERR1740130	SO_SRF_SMALL_ERR1740130	Southern Ocean
ERR1740133	SO_SRF_SMALL_ERR1740133	Southern Ocean
ERR1711918	MS_SRF_SMALL_ERR1711918	Mediterranean Sea
ERR1711995	MS_SRF_SMALL_ERR1711995	Mediterranean Sea
ERR1711998	MS_SRF_SMALL_ERR1711998	Mediterranean Sea
ERR1712006	MS_SRF_SMALL_ERR1712006	Mediterranean Sea
ERR1712022	MS_SRF_SMALL_ERR1712022	Mediterranean Sea
ERR1719164	MS_SRF_SMALL_ERR1719164	Mediterranean Sea
ERR1719224	MS_SRF_SMALL_ERR1719224	Mediterranean Sea
ERR550386	MS_SRF_SMALL_ERR550386	Mediterranean Sea
ERR550396	MS_SRF_SMALL_ERR550396	Mediterranean Sea
ERR550403	MS_SRF_SMALL_ERR550403	Mediterranean Sea
ERR550404	MS_SRF_SMALL_ERR550404	Mediterranean Sea

All analyzed samples were collected from surface water

Despite this performance, some genus-level annotations were missing based on the contigs provided from the MMETSP. Between all trials, an average of 1.3 ± 1.9 genera out of an average of a total 6.1 ± 2.7 genera were not recovered by the *euk*rhythmic re-assembly, despite being present in the MMETSP transcriptomes which were used to create each community (see Table 2). As many of these annotations were also missing from the EUKulele annotations on the contigs from the MMETSP themselves (1.9±1.9 genera), these contigs may simply have not been sufficiently distinct from the transcriptomes of other organisms in the database to be annotated, potentially due to sequence length or specificity. Using the EUKulele annotations instead of the taxonomic annotation of the transcriptome from which the original contigs were taken, $2.8\pm 1.7$ genera were not found in the *euk*rhythmic reassembled results as compared to the original EUKulele annotations of the designer assemblies. An average of $39.3\pm 12.9$ distinct genus-level annotations were assigned in the *euk*rhythmic output as compared to $6.1\pm 2.7$ distinct MMETSP genera being used to generate the samples due to being successfully annotated as similar genera present in the MMETSP. These spuriously taxonomically annotated contigs constituted both a minority of the total assembled contigs as well as estimated abundance from the simulated raw reads (Additional file 1: Fig. S6), and the occurrence of these spurious annotations could be reduced with more stringent EUKulele parameters, though at the expense of some correct annotations.

**Fig. 5:**
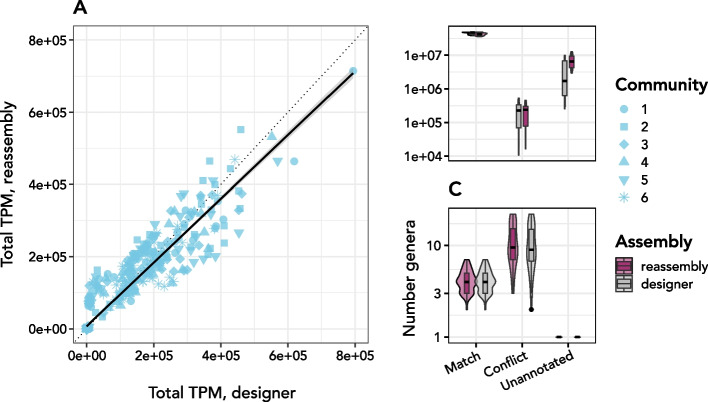
*euk*rhythmic reassemblies accurately recapitulate taxonomic information. **A** summed transcripts per million (TPM) as reported from Salmon mapping of the designer assembly compared to the *euk*rhythmic reassembly. Each point represents a genus; the dotted line is a 1-to-1 line ($y=x$), or collection of reference transcriptomes from the MMETSP. Circle size corresponds to community type (1–6) as described in the text; of note is that some communities have very highly abundant genera, such as the smallest circles corresponding to Community 1. **B** Sum of total TPM in the designer vs. reassemblies that corresponded to genera which (1) matched genera from the original MMETSP transcriptomes, (2) conflicted or did not match genera from the original MMETSP transcriptomes, or (3) were not annotated, according to EUKulele. **C** The number of genera that matched (true positives), did not match (false positives), or were unannotated (false negatives according to database precision). As shown in panel B, unannotated contigs at the genus level were more abundant in the reassemblies than the designer metatranscriptomes. There were also statistically significantly more matches in the designer metatranscriptomes than the reassemblies from *euk*rhythmic. However, false positives were occurred at a similar rate between the two assembly types, indicating that these were more likely a product of the original quality of the contigs from the MMETSP or their ability to be uniquely classified

The sequence annotations were classified according to whether they did or did not align with genus-level annotations from the MMETSP (Fig. 5). There was no statistically significant difference between the per-sample summed abundance of incorrectly-annotated contigs between the designer and the *euk*rhythmic reassembled products (T=−0.084; *p*=0.93), however correctly-annotated contigs were significantly more abundant in the designer assemblies (T=−5.28; *p*=8.3e−7) and unannotated contigs were significantly more abundant in the *euk*rhythmic reassemblies (T=5.43; *p*=4.5e−7).

### Functional annotations from metatranscriptome assembly match abundance and diversity of functions in designer transcriptomes

**Fig. 6:**
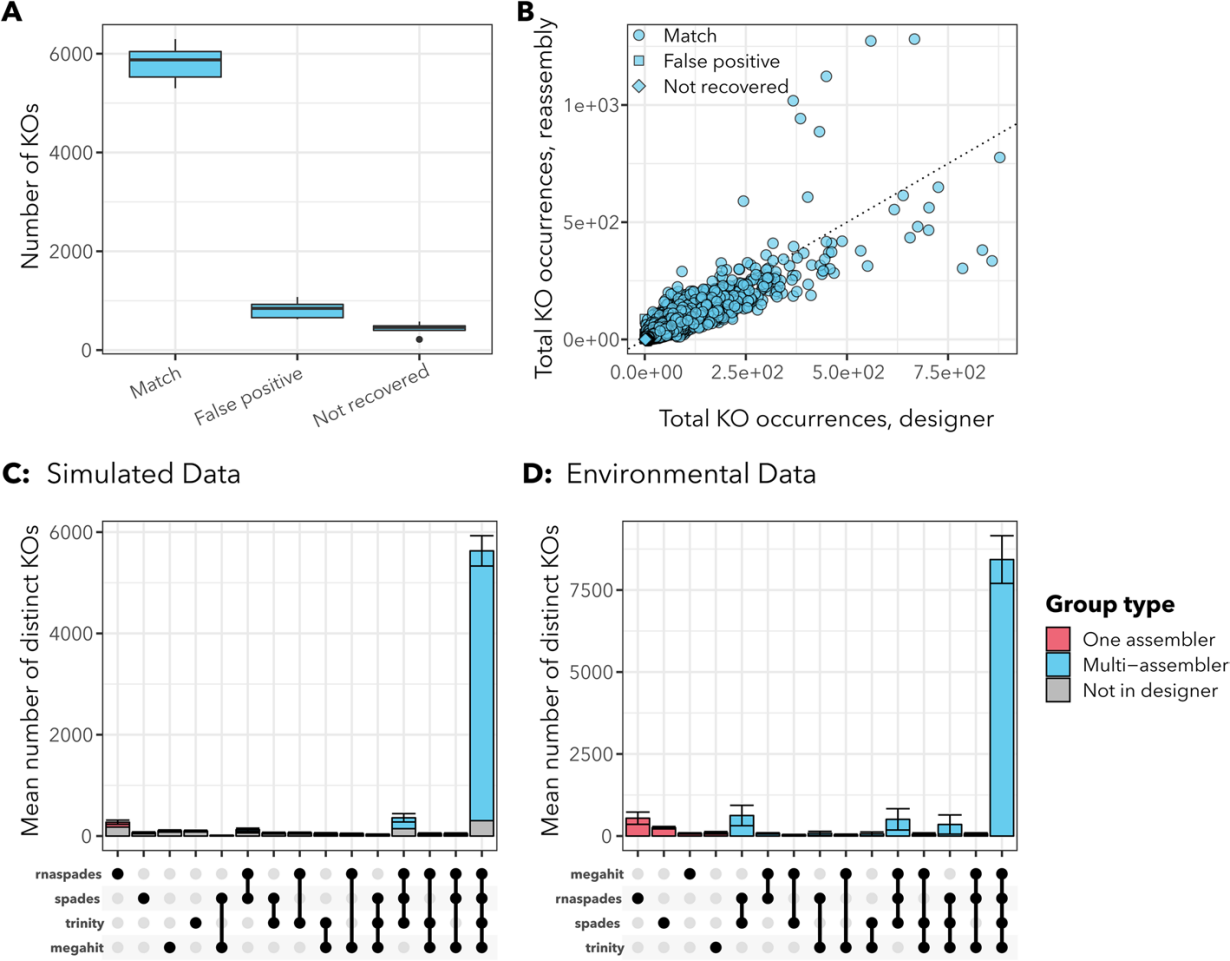
Functional annotation findings from *euk*rhythmic reassembly of the simulated raw reads from the designer metatranscriptomes. **A** summary of KO recovery, showing the total number of KOs that are recovered by the *euk*rhythmic reassembly that were present in the original transcriptomes “Match”, those that were in the *euk*rhythmic reassemblies that were not present in the original designer set “false positives”, and those that were present in the designer assemblies but not recovered by *euk*rhythmic “not recovered”. **B** the number of occurrences of each KO is compared between the designer metatranscriptomes (horizontal axis) and the *euk*rhythmic reassemblies (vertical axis). The dotted diagonal line indicates the one-to-one line. **C** this incidences of each KO in the designer assemblies and the *euk*rhythmic reassemblies are broken up by the individual assemblies that each KO was recovered from (“incidence count” is the number of KOs meeting each category). The majority of all recovered KOs are shown to be recovered by all four assemblers as well as present in the designer metatranscriptomes. Portions of the bar colored in gray indicate that these KOs were recovered by all of the assemblers listed, but were not found in the designer assembly. **D** Environmental data for KOs from Narragansett Bay as a comparison to Panel C

Functional annotations were recovered with similar frequency and relative abundance in the *euk*rhythmic reassembled products as compared to the designer assemblies (Fig. 6; Additional file 1: Fig. S7), and also between assemblers (Additional file 1: Fig. S13). As an overall average across MMETSP groups and samples, 5820.6±349.6 KEGG orthology terms (KOs) were correctly recovered from the designer assemblies, 820.3±163.7 were “false positives” that were recovered in the *euk*rhythmic reassemblies but not in the original designer assemblies, and 473.8±107.6 were identified in the designer assembly but not recovered by *euk*rhythmic. However, the false positive and unrecovered KOs tended to have low abundance compared to those which were correctly identified: on average, there were 1566.5±321.3 total occurrences of annotations of false positive KOs per sample in the *euk*rhythmic reassemblies and 107.6±204.7 total occurrences of annotations of KOs that were not found in the *euk*rhythmic reassemblies in the designer assemblies, as compared to and average of 132751.9±10176.5 occurrences in the designer assembly and 116489.5±9961.0 occurrences in the *euk*rhythmic reassemblies of KOs that were mutually recovered before and after the reassembly process. A linear regression with an imposed *y*-intercept of zero as calculated in R [68] revealed a relationship of $\text {Reassembled KO abundance} = \text {Designer KO abundance} \cdot 0.96$ with an adjusted $R^2$ of 0.85 ($p=2.2e^{-16}$), indicating a nearly one-to-one relationship between the abundances of each KO in the designer assembly and in the reassembled products (including false positives and KOs missing from the *euk*rhythmic reassemblies; Fig. 6).

**Fig. 7:**
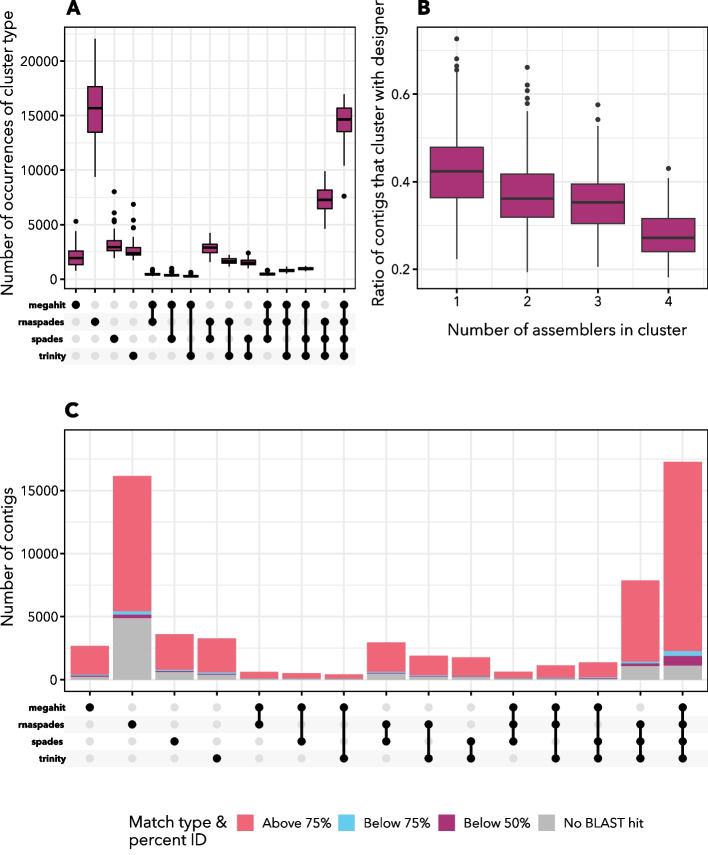
mmseqs2 clustering in *euk*rhythmic collapses redundant sequences and highlights between-assembler differences in fidelity of recovered proteins to designer proteins. Panel **A** The total number of contigs per cluster as separated by the assemblers from which they were recovered. rnaSPAdes produced the highest number of contigs overall independently, which was a higher overall number than the contigs which were produced by all four assemblers (far right boxplot in panel A). Panel **B** The proportion of mmseqs2 clusters of proteins that did not cluster with proteins from the designer assembly as a function of the number of assemblers represented within the cluster. Protein products supported by assembly by all four assemblers were least likely to be “spurious”, or not recoverable from the designer assembly. Panel **C** Number of contigs that had no protein ORF assigned to them via TransDecoder (black) as compared to contigs with proteins having BLAST matches according to some percentage identity. The first stacked bar corresponds to contigs that both had a detected ORF and a BLAST match with percentage identity >75% at an e-value threshold of $10^{-2}$. Additional file 1: Fig. S11.4 shows the contigs from the designer assembly which originally did not have an identified ORF

The vast majority of KOs also recovered in the designer assemblies were identified by all four assembly tools (5326.6±247.9 KOs across all samples). rnaSPAdes individually recovered the highest number of unique KOs that were also found in the designer assembly of any assembler (96.0±20.4), but rnaSPAdes also generated the highest number of KOs that were not found in the designer assemblies (176.4±26.5), nearly double the number that it uniquely recovered (Fig. 6). rnaSPAdes also had both the highest number of proteins that did and did not have a successful BLAST ([73, 78, 79], Fig. 7).

### Applying the eukrhythmic pipeline to environmental metatranscriptomic datasets

**Fig. 8:**
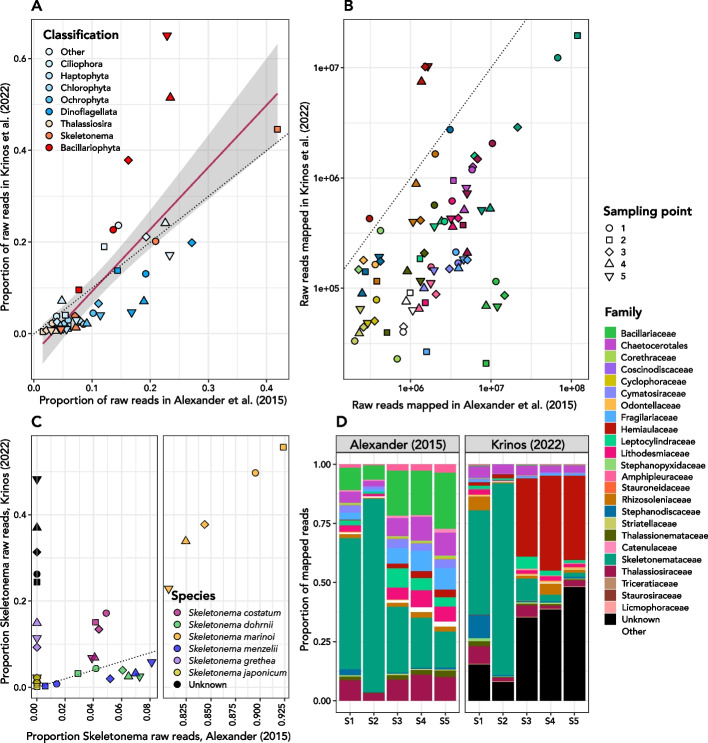
Narragansett Bay dataset from Alexander et al. (2015) [23] assembled using *euk*rhythmic. **A** The correspondence between the proportion of total raw reads in (y) this study vs. (x) [23]. Each point represents a sampling time, and *Bacillariophyta* aggregates all non-*Skeletonema* and non-*Thalassiosira* diatoms. **B** Family-level taxonomic breakdown of [23]’s raw read mapping (left) as compared to this study. **C** Log-normalized raw reads mapped to each taxonomic family compared between the two studies. **D** *Skeletonema* species represented in the *euk*rhythmic reassembly representing some of the diversity within this genus known to show seasonal dominance in Narragansett Bay

To benchmark the *euk*rhythmic pipeline and to provide examples of the potential biological insights that can be extracted from the assembly approach, we assembled and annotated samples from two metatranscriptomic datasets. First, we chose two sets of samples from the *Tara* Oceans project as a representative general oceanographic dataset: one set from the Southern Ocean and one from the Mediterranean Sea, two ocean basins with contrasting levels of diversity (Fig. 8A–C). We found that *euk*rhythmic expands the total amount of protistan coding sequence data that can be recovered from anywhere in the global ocean. We also assembled a metatranscriptomic dataset from a previously published study in Narragansett Bay as a coastal example with a dominant taxonomic group (i.e. a canonical “bloom” scenario). We observe that while *euk*rhythmic recapitulates many of the general patterns from a direct read mapping-based study, the assembly approach outperforms direct read mapping with respect to the number of distinct diatom representatives recovered, the dominant taxonomic group (*Bacillariophyta*) in the samples.

#### *Tara* Oceans eukrhythmic assemblies contain coding sequences that lack representation in the “MATOU” gene atlas

More than one in six (17.5%) of the coding sequences we recovered from our multi-assembler *euk*rhythmic assemblies from the *Tara* Oceans metatranscriptomes had no significant hits to the “MATOU” composite gene atlas across all *Tara* Oceans metatranscriptomes curated by [22] (Fig. 8D-F; percentage mapping of these coding sequences to the raw reads as compared to all contigs shown in Additional file 1: Fig. S20). An average of 16.1% of all Mediterranean Sea coding sequences and 18.8% of all Southern Ocean sequences did not have any match to previously recovered coding sequence content in the MATOU database, which includes coding sequences from all major global ocean basins. These results indicate the expansion of coding sequences achieved by using *euk*rhythmic, but also that total number of coding sequences is not evenly expanded across samples (Fig. 8E); while in some samples >75% of coding sequences did not have a match to the MATOU database, in others it was <10%.

As much as 41.3% of the coding sequence products of the assembly for each sample could not be assigned a taxonomic annotation via EUKulele, but more so in samples from the Mediterranean Sea (Additional file 1: Fig. S21); mean Mediterranean Sea: $34.8\pm 5.2$%, mean Southern Ocean $28.0\pm 4.4$%). Among the fraction of sequences that had EUKulele taxonomic annotations and were not found in the MATOU database, dinoflagellates dominated the recorded number of coding sequences recovered in both basins (mean Mediterranean Sea: $12.0\pm 7.7$%, mean Southern Ocean: $34.0\pm 10.1$%; Fig. 8F). Dinoflagellates also dominated in terms of average proportion of total TPM (SO: 30.3±11.1%; MS: 8.6±6.6%), but not in terms of mean raw TPM assigned in the Southern Ocean (SO: Ochrophytes had the highest assigned TPM at 32853.1±66098.9, while dinoflagellates had 31853.4±82808.6 assigned TPM), because some samples dominated by dinoflagellates also had a relatively low number of reads assigned to coding sequences not found in the MATOU database (Fig. 8E, F). All of the taxonomic annotation information for the fraction of the *euk*rhythmic sequences that had a EUKulele annotation is summarized in Additional file 1: Fig. S22.

Our efforts expand the total coding sequence content available from global ocean metatranscriptomes, but also highlight the ongoing need for intercomparison of approaches. The average length of coding sequences that did not have a match to the MATOU database was 466.9±243.1 bases, while the average length of the coding sequences that did have a match was 613.5±438.0 bases (Additional file 1: Figs. S23, S24). The average length of the coding sequences with a match (i.e., recovered by both assembly efforts) was significantly longer (*t*=720.86; $p<$1e−16). The use of a *k*-mer size of 63 with the velvet assembler by [22] may also have contributed to this result: the rnaSPAdes assembler, for example, takes into account the varying coverage level of expression data by using a *k*-mer size that varies dynamically with read length [36]. This approach occasionally increases misassembly rate, but also protects rarely-expressed genes from being missed [36]. Because eukaryotic communities in the microbial ocean may be sparse and contain rare taxa, we argue that a more exhaustive assembly approach is warranted, even if the average length of assembled sequences is reduced.

#### *Tara* Oceans assembly raw read fidelity and non-coding sequences

*Tara* Oceans assemblies from the Mediterranean Sea and Southern Ocean varied in their overall composition as well as the accuracy of their recovery in the assembly process. While we focused the balance of our analysis on predicted coding sequences to compare to the analysis by [22], we note that via Salmon mapping, an average of 30.1±10.7% of the raw reads for the Mediterranean Sea samples mapped back to the coding sequences, as compared to 51.5±13.3% for the full assembly, while in the Southern Ocean samples, 51.5±11.6% of the raw reads mapped back to coding sequences as compared to 76.4±10.3% for the full assembly (Additional file 1: Fig. S22). This indicates that in both cases, a substantial fraction (>20%) of the original raw reads can be assembled into contigs, but appear to be non-coding. These non-coding sequences may be involved in important regulatory processes [80, 81], such as nutrient stress in diatoms [82], hence should not be excluded from consideration.

#### Multiple assemblers improve metatranscriptome assembly of Narragansett Bay phytoplankton

**Fig. 9:**
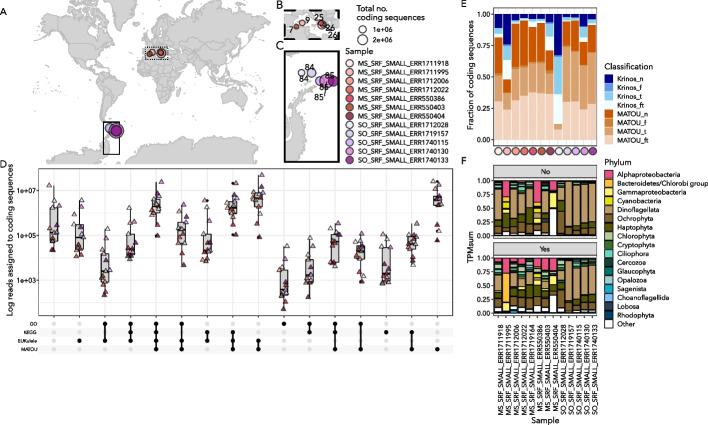
*Tara* Oceans reassemblies using *euk*rhythmic (Carradec et al. (2018) [22]). **A** Map showing the locations of reassembled *Tara* Oceans samples. Boxes over regions are expanded in Panels B and C. **B** Mediterranean Sea samples. Numbers indicate *Tara* Oceans stations. **C** Southern Ocean samples. As in Panel B, numbers indicate *Tara* Oceans stations. **D** between-assembler overlap of the reads assigned to coding sequences. The x-axis indicates the annotations assigned to each of the coding sequences, and the y-axis shows the between-sample sum of reads assigned to coding sequences for that category. **E** Fraction of coding sequences that did or did not have a match to the MATOU database. Shades of blue indicate coding sequences recovered only by this study. The top segment indicates coding sequences without functional or taxonomic annotations, following by the proportion of sequences with functional and taxonomic annotations (“ft”), the proportion with only functional annotations (“f”), and the proportion with only taxonomic annotations (“t”). The same is shown in shades of orange for the assembld coding sequences from this study that did have a significant match to the MATOU database. The y-axis shows the color-coded *Tara* Oceans sample. F: The fraction of TPM assigned to coding sequences with recovered taxonomic annotations. These are from the “Not in MATOU” “ft” and “t” bars in Panel E. Dinoflagellated dominate many of the Southern Ocean samples, particularly for those coding sequences which could not be taxonomically annotated

We benchmarked the *euk*rhythmic pipeline using a previously analyzed marine metatranscriptomic dataset [23] (Fig. 9). In particular, we were able to recapitulate the taxonomic composition of the diatom-dominated community described in [23]. Across all assemblers, representative from the phylum *Ochrophyta* were suggested to be dominant members of the community (Fig. 9A, D; Additional file 1: Fig. S19), and moreover the genera *Skeletonema* and *Thalassiosira* were recovered in expected proportions, with *Skeletonema* producing a numerically-dominant bloom determined via cell counts obtained from microscopy in sample S2 (Additional file 1: Fig. S15). Notably, our assembly recovered a greater diversity of diatom species than the raw read mapping method used previously (Fig. 9; [23]), including the recovery of multiple species of *Skeletonema* known to be present in this ecosystem ( [83]; Fig. 9D).

While broad patterns in taxonomic annotations were indistinguishable between the different assemblers and the majority of KEGG Orthology (KO) IDs were recovered by all four assemblers (Fig. 9B, C), the assemblers showed some differences with respect to the abundance of each functional annotation. In particular, MEGAHIT reported fewer instances of each functional gene grouping than rnaSPAdes, and fewer than Trinity approximately half of the time (Fig. 9; Additional file 1: Fig. S12). rnaSPAdes appeared to report a lower overall abundance of diatoms when the normalized TPM metric returned by Salmon [71] was used, but this pattern did not hold when non-normalized raw reads were used instead (Fig. 9; Additional file 1: Figs. 14, 15, 16). Contigs generated by the assemblers that were successfully annotated as *Skeletonema* or some other diatoms appeared to have longer average length than the average among all taxa (mean length of *Skeletonema* contigs with standard error of the mean: $618.6\pm 0.7$; overall mean: $396.6\pm 0.07$; two sample t-test t = 310.17 $p<2.2e-16$; Additional file 1: Fig. S18). rnaSPAdes produced a disproportionately high number of contigs relative to the other assemblers, many of these contigs belonging to non-diatom taxa (Additional file 1: Fig. S18). These contigs also tended to be shorter in the rnaSPAdes assemblies, both for non-diatom taxa (rnaSPAdes mean ± standard error = $377.7\pm 0.1$; overall mean = $421.1\pm 0.09$; t-test t=−367.17, $p<2.2e-16$) and for unannotated contigs (rnaSPAdes mean ± standard error = $264.7\pm 0.7$; overall mean = $300.8\pm 0.07$; t-test t=−498.5, $p<2.2e-16$). While these differences were universal for annotated non-diatom and unannotated contigs, rnaSPAdes produced shorter *Skeletonema* contigs than Trinity (t=−101.1; $p<2.2e-16$), but longer *Skeletonema* contigs than both MEGAHIT (t=41.6; $p<2.2e-16$) and SPAdes (t=64.0; $p<2.2e-16$).

## Discussion

Metatranscriptome analysis has become a widespread approach for extracting taxonomic and functional information from protistan communities across a variety of environments ranging from coastal to open ocean marine ecosystems to soil ecosystems [12–14, 23, 69, 84]. Here, we designed a multi-assembler pipeline for metatranscriptomic assembly, *euk*rhythmic, and evaluated its performance on both simulated metatranscriptome data from the MMETSP [20] as well as on previously published metatranscriptome datasets [22, 23]. In doing this, we explored the relative performance of commonly-used assemblers, and determined that a multi-assembler approach improves the outcomes of metatranscriptome assembly with regard to recapitulating proteins and their taxonomic and functional annotations.

### Scalable and reproducible pipelines like eukrhythmic enhance intercomparison and advance computational research

*Euk*rhythmic enables the simultaneous processing of many metatranscriptomes at once, and its modular design allows for reproducibly reprocessing the results of previous analyses as new tools become available. As datasets become larger, research questions now aim to tackle ambitious questions across space and time scales. Increasingly complex datasets necessitate careful workflow management [85, 86]. We have developed a pipeline that manages large metatranscriptomic datasets with the goal of assessing the diversity and function of marine protists, and have demonstrated the usefulness of our modular tool via the new insights the tool draws from previously analyzed, published metatranscriptomes [22, 23]. In particular, the reanalysis of data from [22, 23] highlights the expanded analytical insights that might be derived from a multi-assembler approach such as that provided by *euk*rhythmic, and these datasets can continue to be reproducibly re-analyzed with *euk*rhythmic as improved assembly tools become available. Further, despite the roughly sixteen discrete software steps that occur during a run of *euk*rhythmic, processes are run in parallel and can be deployed to the cluster, meaning that reanalysis that would ordinarily take multiple days per sample could now take the same amount of time for the entire project, resource-dependent.

### Do metatranscriptomes capture the diversity of protistan communities?

Environmental metatranscriptomes are a community mosaic of ephemeral RNA-based signals of expression. Metatranscriptomes are increasingly a routine diagnostic tool for making important conclusions about community composition and function within marine systems [12–14, 23, 69, 87, 88], and are being applied to establish comparison at global scales [22, 69] and over long time periods [89–91]. Despite this, best practices for physical collection, molecular processing, and bioinformatic analyses have yet to be established [86]. Towards the standardization of computational approaches to marine protistan metatranscriptomics [86], we have shown that a multi-tiered eukaryotic metatranscriptome assembly pipeline recapitulates annotated contigs from a mock transcriptome community. Notably, the contigs produced from multiple assemblers tend to be of the highest quality with regard to their similarity to the original contigs from the transcriptome assemblies via clustering, taxonomic, and functional annotations. We find that metatranscriptomic approaches to assess community diversity and function in the environment are indeed adequately and reproducibly recapitulating the taxonomic and functional diversity of the RNA pool and of those environments when they use assembly approaches similar to that employed by *euk*rhythmic.

Our reassembly of environmental metatranscriptomic datasets further highlights the power of the multi-assembler approach in recovering novel gene content. In the samples from diatom-dominated Narragansett Bay [23], we recovered a greater diversity of diatoms than raw read mapping alone in the original analysis, a level of diversity which aligns with other studies from the region [83]. From the *Tara* Oceans samples, we found novel protein sequences not recovered and included in a comprehensive, global analysis effort using a single assembler [22], more than half of which had functional and/or taxonomic annotations. Even when the final coding sequences were clustered and only contigs of sufficient length were retained following [22], all samples contained previously unknown coding sequences, and some samples contained more unknown than known sequences. Though not all of these coding sequences could be annotated, recent progress has been made towards annotating genes of unknown function [92], which can be highly abundant in metatranscriptomic data. These results demonstrate the value in reassembling previously analyzed datasets using multiple tools with different underlying algorithms.

One important note is that even the best resource that we have available for consensus-based taxonomic annotation of *de novo* mixed community metatranscriptome assemblies constrains our efforts before we begin: laboratory-derived sequenced transcriptomes of single organisms cannot be fully reverse-annotated. In other words, even when we use sequence search tools to recover the taxonomic annotation of a contig present in the database, some of these sequences are too short or share a non-negligible percentage of sequences between organisms and cannot be annotated to a fine level of resolution, even in their unmodified state. In these cases, the fact that we can recover many, but not all, of originally annotated contigs after reverse-engineering the community tells us more about the limits of taxonomic annotation via short-read sequences than it does about the pitfalls of the assembly process. Hence, it is critical that we continue to consider the shortcomings of the annotation process as we analyze and re-analyze metatranscriptomic datasets.

### Is there one best assembler for eukaryotic environmental metatranscriptomes?

An additional goal of our analysis was to compare the performance of different assemblers on eukaryotic metatranscriptome data, and to determine whether the use of multiple assemblers is warranted. According to our results, no single assembler we assessed (MEGAHIT [47], rnaSPADes [36], metaSPAdes [48], and Trinity [61]) is universally the best choice. *De novo* sequence assembly has both technical and practical considerations. Beyond simply balancing run time, memory requirements, and optimal accuracy, the performance of assemblers is difficult to evaluate. In particular in community assembly, low sequencing depth may complicate typical approaches used to reduce the effects of sequencing error. In our study, two assemblers stood out as the bookends of the spectrum of assembly approaches. MEGAHIT produced long contigs, but had the lowest percentage mapping of raw reads to the assembly, while rnaSPAdes routinely had the highest raw read mapping percentage and number of functional annotations (Fig. 9; Additional file 1: Fig. S12), but had shorter contigs on average, and a high incidence of transcripts that did not appear to be coding. These patterns held in both simulated and environmental datasets (Figs. 3 and 6; Additional file 1: Figs. S2–S5).

The spectrum of approaches adopted by assemblers also had a significant impact on the interpretation of assembly products. This effect may be clearest when considering how the average length of contigs recovered by an assembler (e.g. MEGAHIT and rnaSPAdes as in the example above) can directly skew interpretation of community composition. Shorter transcripts will recruit fewer reads, but will appear more abundant when a normalization that takes sequence length into account is used [93]. Because assemblers that work like rnaSPAdes produce a greater number of shorter contigs that may not be annotated, organisms or individual contigs or predicted genes with longer transcript length appear comparatively less abundant when reads are normalized, but not when non-normalized raw reads alone are used (Additional file 1: Figs. S15, S16, S17). For example, in the Narragansett Bay samples, we observe that diatom *Skeletonema* appears to have higher mean contig length - at least in the contigs which can be accurately assembled and taxonomically labeled by the EUKulele tool. However, conventional community composition metrics like TPM that normalize to contig length will penalize the recruitment of raw reads to these longer-than-mean contigs. Yet, as has been well described for transcriptomes, using raw reads leaves interpretation vulnerable to biases related to sequencing depth, sequencing approach, and transcript length, intuitively because longer transcripts are expected to recruit a greater number of raw reads by virtue of their size [93]. In a mixed community sample, and particularly in marine communities in which organisms are extraordinarily diverse, normalizations need to take the heterogeneity of the community into account.

Taken together, these results support the potential utility of merging the subtly different approaches taken by different assembly tools, in order to maximize gene recovery whilst also retaining the distinct signatures that make community composition interpretable. rnaSPAdes-like approaches improve functional recovery, while MEGAHIT-like assemblers produce longer sequences, which possibly have higher fidelity to the observed community. This observation further raises the question of how we can or should be extracting community composition insights from metatranscriptomes, especially when samples cannot or have not been normalized to housekeeping or spike-in sequences.

### Should we be downsizing metatranscriptomic assemblies?

Computational constraints continue to limit the scale of metatranscriptomic analyses, as downstream tools for e.g. abundance quantification and functional annotation may have sizable memory requirements for excessively large assembly files [94]. Here, we advocate for a multi-assembler approach to metatranscriptome assembly. As we have discussed, the multi-assembler approach generates a greater number of total predicted coding sequences, and many of the additional coding sequences assembled from our simulated dataset are similar taxonomically, functionally, and via sequence identity to coding sequences from the designer assembly (Figs. 5, 6, 7; Additional file 1: Fig. S8). However, using a multi-assembler approach will create larger assemblies, and users need to be cognizant of the complexity of their dataset and memory usage requirements downstream. Researchers may address excessive assembly size by (1) intentionally limiting the assembly to downsized, high-quality content, or (2) by more strictly clustering assembly products, the choice between which comes down to the research question.

The contigs statistically most likely to contain detectable open reading frames and to closely resemble “true” sequence content in a real-world sample via blast sequence search and mmseqs2 clustering are those that multiple assemblers can agree upon (Fig. 7; Additional file 1: Figs. S8–S11). Researchers may choose to maximize confidence in assembly products by using only the contigs discovered by more than one assembler, or may expand the total number of recovered genes by leveraging multiple algorithms. While the intention of *euk*rhythmic is to combine the outputs of multiple independently-contributing tools, in an analysis in which the goal is to extract only the products which can be assumed to be of the highest quality, the smaller intersection between assembly tools may be retained. This would also substantially reduce the number of sequences, improving the computational feasibility of downstream analyses. For example, if a researcher was interested specifically in generating a core set of high-confidence genes for a site and then mapping raw reads back to a combined assembly to detect changes in expression over time and space, multiple assembly may furnish a set of transcripts more likely to both be accurately retrieved from the original samples as well as ecologically relevant for mapping. However, it is important to note two pitfalls of this approach. First, this substantially decreases the proportion of the raw reads that are represented in the final contigs after assembly (Fig. 7). Secondly, while some assemblers produce a higher number of sequence products that *do not* have detectable similarity to the “true” contigs from which raw reads were simulated, they also produce a number of *unique* sequences that *are* detectable in the original assembly and, crucially, not identified by any of the other assembly approaches (e.g., rnaspades).

Researchers may also opt to cluster the resulting assembly in accordance with their desired final file size or level of sequence redundancy. Using mmseqs2 clustering [50], we found that for our combined assemblies, the choice of clustering parameters is an important one, with potentially significant reductions in file size being possible without appreciable impact on the functional and taxonomic profile of the assembled metatranscriptome (Fig. 4; Additional file 1: Fig. S1). Approaches are now being developed to more reliably and efficiently cluster predicted genes of both known and unknown function, for example using tools like mmseqs2 coupled to functional domain information and probabilistic modeling [92]. Such approaches are particularly useful for fitting coding sequences from a single assembly into the context of expansive datasets in space and/or time, from which many millions of total coding sequences can be extracted, and computational processing becomes exceptionally limiting [92].

### eukrhythmic: an approach for optimized multi-assembler metatranscriptome assembly

Metatranscriptome quality cannot be assessed using either genomic or single-organism metrics. Instead, the assembly products need to be considered as potentially novel genetic content when assessing assembly success. Here, we present *euk*rhythmic, a workflow for assembling environmental metatranscriptomes of eukaryotic communities by leveraging multiple assemblers. We evaluate our pipeline both using existing environmental metatranscriptomes and simulated community data that we generated using a second pipeline, jEUKebox. The flexible jEUKebox pipeline can be reused as additional reference sequences become available in order to test community ecology hypotheses for cultured and uncultured organisms. Simulating communities and testing their ability to be recovered is an essential step in ensuring the fidelity of metatranscriptome studies as the volume of taxonomic and functional data available to make predictions grows. In particular, we envision the construction of metacommunity data using uncultured organisms inferred from metagenomic sequences (metagenome-assembled genomes (MAGs)) [10]. Our inability to annotate some unmodified contigs from the original simulated community highlights crucial questions about the limits of annotation. Are some genes destined to remain difficult to annotate (taxonomically and functionally), either because they vary too dramatically between organisms, hence an organism-specific and highly complete genome is needed to accurately identify them, or because they are part of an indistinguishable cluster of highly similar genes? Can we be sure that these are true genes, or could they be artifacts of the assembler originally used to generate the reference assemblies? Rigorous simulations of communities may help to identify these difficult-to-annotate genes and to set thresholds that prevent erroneous annotations, in conjunction with new approaches for annotating unknown genes [95]. Computational simulations should be paired with laboratory curation of cultivated communities and accompanying metatranscriptomic sequencing which can be compared with count data. Promising plans for executing these steps are already in place [96].

Critical assessment of the accuracy and quality of metatranscriptome assembly and quantification of technical impacts, such as clustering similarity or the algorithms used to construct contigs, provide confidence for ecological interpretations. The *euk*rhythmic pipeline represents a reproducible roadmap towards assembling new eukaryotic environmental metatranscriptomes, and reassembling the growing repository of existing eukaryotic environmental metatranscriptomes with multiple assemblers. This flexible tool that researchers can use to standardize the crucial steps of metatranscriptome analysis is a step towards the standardization and validation eukaryotic metatranscriptome assembly. With the consistent use of software tools and pre- and post-processing steps that *euk*rhythmic enables, metatranscriptome assembly has the potential to unlock the functional roles of largely uncharacterized eukaryotic microbes that drive biogeochemistry across diverse natural ecosystems. Standardized workflows for eukaryotic metatranscriptome assembly like *euk*rhythmic and community simulations as powered by tools like jEUKebox are a vital means to validate these discoveries.

## Supplementary Information

**Additional file 1: Fig. S1.** Visual summary of the effect of clustering with mmseqs2 on recovered enera, KOs (functional annotations) and file size in bytes. While reducing the equence identity threshold for clustering results in up to a 30% average reduction in file size, he number of recovered genera and functional annotations are only modestly impacted, specially at high coverage. An intermediate sequence identity of 0.8 and coverage of .8 would result in a 15-25% average reduction in file size, but leave distinct functional and taxonomic annotations unchanged.** Fig. S2.** Main text figure facetted by simulated METSP assembly group (two different sets of organisms). Of note is that the *euk*rhythmic reassembly accurately recapitulates the bimodal distribution in GC-content observed in MMETSP group A’s designer metatranscriptomic sequences.** Fig. S3.** Protein sequence lengths in the reassemblies as compared to the designer. The 1-to-1 line shows where sequences would fall if the average length of recovered protein sequences via TransDecoder were identical between the designer assemblies and the *euk*rhythmic-derived reassembled products; the fact that all samples fall in the lower right half of the plot indicates that protein sequences were consistently larger in the designer assemblies as compared to the *euk*rhythmic reassembled products.** Fig. S4.** Mean contig length as a function of the number of assemblers that found a sequence that matched the given description. For the 4-assembler cluster, this means that all four assemblers tested identified a sequence that matched the sequence included in the distribution when clustered within *euk*rhythmic. Panel A corresponds to MMETSP Group A while Panel B corresponds to MMETSP Group B. Welch’s independent T-tests and Kolmogorov-Smirnoff tests for between-distribution goodness of fit computed on these length distributions reveals that the overall distribution of lengths for 1 vs. 2 vs. 3 vs. 4-assembler distributions are all statistically significantly different (p < 1e−6), with greater assemblers within a cluster leading to higheraverage length.** Fig. S5.** Salmon mapping percentages against the simulated raw reads when computed individually against each of the four assembly tools used by *euk*rhythmic. rnaSPAdes consistently outperformed the other assemblers with regard to percentage mapping, average length, and number of annotations.** Fig. S6.** Main text figure, but with A: TPM comparison for the designer assemblies and the reassembled products from euk rhythmic labelled by distinct groupings of simulations relative to their subset of MMETSP organisms; each point is filled according to its “MMETSP group”.** Fig. S7.** Main text figure with the results split by “MMETSP group” to demonstrate that different taxonomic groupings of organisms included in the simulation does not impact the general trends observed in the results. All three panels are divided by samples using the two “MMETSP groups” of individual organism transcriptomes.** Fig. S8.** Salmon percentage mapping by MMETSP group when contig products from all assemblers as in *euk*rhythmic are used (bottom distribution with one or more assembler) as compared to when only contigs agreed upon by multiple assemblers are used. Average percentage mapping decreases progressively with fewer contigs being included as the criteria for inclusion are made stricter.** Fig. S9.** Proportion of contigs from each clustering subset that had ORFs extracted from the sequence. The vast majority of contigs had a single predicted ORF, but rnaSPAdes alone had the greatest number of nucleotide contigs on which an ORF could not be detected. In practice, these sequences might be assumed to be non-coding.** Fig. S10.** Proportion of contigs from each clustering subset that had ORFs extracted from the sequence across all contigs identified by the assembler. rnaSPAdes had a higher number of contigs without an identified ORF.** Fig. S11.** Proportion of contigs from the designer assembly that had ORFs extracted from the sequence. In general, fewer contigs did not have an identified ORF, and a higher number of contigs had multiple predicted ORFs than in the reassembled products from euk rhythmic.** Fig. S12.** Comparison of the abundance of KO IDs within functional annotations across Narragansett Bay samples and different combinations of metatranscriptome assemblers. A black dotted line indicates a one-to-one relationship, meaning that the abundance of KOs that fall along this line are exactly as abundant using the assembler listed on the x-axis and using the assembler listed on the y-axis. On the top left, Trinity is compared to MEGAHIT, on the top right Trinity is compared to rnaSPAdes, on the bottom left MEGAHIT is compared to rnaSPAdes and on the bottom right MEGAHIT is compared to SPAdes. Each point corresponds to a single KO within a sample. While rnaSPAdes tended to report high abundances of each identified KO relative to the other assemblers, MEGAHIT reported fewer instances of each KO than the other three assemblers in most samples. This may be due to the approaches adopted by the two assemblers. Whereas in a typical assembly, k-mers that appear only once are assumed to be the result of error, these k-mers may represent real and important diversity in a set of low-abundance whole-community sequences [47]. The MEGAHIT assembler is an example of metagenomics-specific software that defines “mercy k-mers” which come into play in between two k-mers within a single read that are sequenced more than once. rnaSPAdes, as an example, does not employ a “mercy” strategy but instead decreases the coverage threshold significantly in comparison to genomic assembly [36]. One is adopting the meta-omic assumption that exceedingly low coverage is plausibly not artificial, while the other is being more generous with respect to coverage and memory use, but intending merely to minimize the influence of sequencing error. The strategy adopted by MEGAHIT may result in incomplete consideration of isoforms, which could have contributed to the relatively low recovery rate of multiple copies of the identified KO groups by the MEGAHIT assemblies of the Narragansett Bay metatranscriptome samples.** Fig. S13.** Proportion of total normalized TPM value of major taxonomic groups according to the four metatranscriptomic assemblers that were tested. rnaSPAdes had a lower proportion of TPM assigned to Ochrophyta (including diatoms), but further investigation appeared this to be in large part a consequence of the large number of small contigs produced by rnaSPAdes.** Fig. S14.** Proportion of total normalized TPM as reported by Salmon from the raw reads assigned to each taxonomic category in the assemblies produced by each of the four assemblers (facets). In particular, in sample S2 the taxonomic breakdown of the eukaryotic community differs importantly in the midst of a bloom of the diatom *Skeletonema*.** Fig. S15.** Proportion of assigned raw reads as reported by Salmon assigned to each taxonomic category for the four metatranscriptome assemblers, expressed as a proportion of the total.** Fig. S16.** Proportion of assigned raw reads as reported by Salmon assigned to each taxonomic category for the four metatranscriptome assemblers, expressed as a proportion of the total.** Fig. S17.** Average contig length by annotation of contigs generated by the four assembly tools across samples. Error bars show the standard error of the mean.** Fig. S18.** Total number of contigs generated by the four tested assemblers for each of the taxonomic groupings considered. rnaSPAdes tended to produce more contigs than the other four assemblers, but these contigs were often shorter and occasionally led to misleading community composition results.** Fig. S19.** Analogous stacked abundance plots by taxonomic grouping to the relative abundance plots presented in Alexander et al. [23] for the five in-situ sampling points collected from Narragansett Bay.** Fig. S20.** Salmon percentage mapping of coding sequences (left) vs. entire contigs (right) for the Tara Oceans samples. Mapping only to coding sequences from the assembly decreased the mean percentage mapped, as reported by Salmon.** Fig. S21.** Per-sample taxonomic annotations for all euk rhythmic CDSs, including those which were and were not found to have a match to the MATOU database.** Fig. S22.** Total TPM assigned per sample to sequences that were assigned a EUKulele annotation, but did not have a significant blast match to the MATOU database from Carradec et al. [22].** Fig. S23.** Full length distribution of coding sequences recovered by the *euk*rhythmic assembly and were (left) not found in the MATOU database [22] vs. (right) found in the MATOU database.** Fig. S24.** Per-sample average length of coding sequences recovered by the *euk*rhythmic assembly and were (left) not found in the MATOU database [22] vs. (right) found in the MATOU database.** Table S1.** (Supplement) Effect of clustering designer assembly on assembly size and annotations. Clustering was performed on the original “designer metatranscriptome” set of contigs from the MMETSP references using the mmseqs2 tool (Mirdata et al. 2019). *euk*rhythmic uses a coverage level of 0.98 and sequence identity of 1 for mmseqs2 clustering. See Supplementary Figure 1 for a graphical summary of the influence of sequence identity and coverage on the size of the recovered assembly and its functional and taxonomic annotations

## Data Availability

All code is available in public GitHub repositories https://github.com/alexanderlabwhoi/eukrhythmic and https://github.com/alexanderlabwhoi/jeukebox. Sample output from *euk*rhythmic for the simulated trials is available at https://osf.io/te7sp/. Code used to generate figures is availability in a public GitHub repository at https://github.com/akrinos/2022-Krinos-eukrhythmic.

## References

[1] Massana R, Pedrós-Alió C (2008). Unveiling new microbial eukaryotes in the surface ocean. Curr Opin Microbiol.

[2] Worden AZ, Follows MJ, Giovannoni SJ, Wilken S, Zimmerman AE, Keeling PJ (2015). Rethinking the marine carbon cycle: factoring in the multifarious lifestyles of microbes. Science.

[3] Caron DA, Alexander H, Allen AE, Archibald JM, Armbrust EV, Bachy C, Bell CJ, Bharti A, Dyhrman ST, Guida SM (2017). Probing the evolution, ecology and physiology of marine protists using transcriptomics. Nat Rev Microbiol.

[4] Caron DA, Worden AZ, Countway PD, Demir E, Heidelberg KB (2009). Protists are microbes too: a perspective. ISME J.

[5] Lawler SP, Morin PJ (1993). Food web architecture and population dynamics in laboratory microcosms of protists. Am Nat.

[6] Stoecker DK (1998). Conceptual models of mixotrophy in planktonic protists and some ecological and evolutionary implications. Eur J Protistol.

[7] Sherr EB, Sherr BF (2002). Significance of predation by protists in aquatic microbial food webs. Antonie Van Leeuwenhoek.

[8] Del Campo J, Guillou L, Hehenberger E, Logares R, López-García P, Massana R (2016). Ecological and evolutionary significance of novel protist lineages. Eur J Protistol.

[9] Del Campo J, Balagué V, Forn I, Lekunberri I, Massana R (2013). Culturing bias in marine heterotrophic flagellates analyzed through seawater enrichment incubations. Microb Ecol.

[10] Alexander H, Hu SK, Krinos AI, Pachiadaki M, Tully BJ, Neely CJ, Reiter T. Eukaryotic genomes from a global metagenomic dataset illuminate trophic modes and biogeography of ocean plankton. bioRxiv. 2021. 10.1128/mbio.01676-23 PMC10746220 37947402

[11] Delmont TO, Gaia M, Hinsinger DD, Frémont P, Vanni C, Fernandez-Guerra A, Eren AM, Kourlaiev A, d’Agata L, Clayssen Q (2022). Functional repertoire convergence of distantly related eukaryotic plankton lineages abundant in the sunlit ocean. Cell Genom.

[12] Gifford SM, Sharma S, Rinta-Kanto JM, Moran MA (2011). Quantitative analysis of a deeply sequenced marine microbial metatranscriptome. ISME J.

[13] Becker KW, Harke MJ, Mende DR, Muratore D, Weitz JS, DeLong EF, Dyhrman ST, Van Mooy BA (2021). Combined pigment and metatranscriptomic analysis reveals highly synchronized diel patterns of phenotypic light response across domains in the open oligotrophic ocean. ISME J.

[14] Salazar G, Paoli L, Alberti A, Huerta-Cepas J, Ruscheweyh H-J, Cuenca M, Field CM, Coelho LP, Cruaud C, Engelen S (2019). Gene expression changes and community turnover differentially shape the global ocean metatranscriptome. Cell.

[15] Stewart FJ, Ulloa O, DeLong EF (2012). Microbial metatranscriptomics in a permanent marine oxygen minimum zone. Environ Microbiol.

[16] John DE, Zielinski BL, Paul JH (2009). Creation of a pilot metatranscriptome library from eukaryotic plankton of a eutrophic bay (Tampa Bay, Florida). Limnol Oceanogr Methods.

[17] Sunagawa S, Acinas SG, Bork P, Bowler C, Eveillard D, Gorsky G, Guidi L, Iudicone D, Karsenti E, Lombard F (2020). Tara Oceans: towards global ocean ecosystems biology. Nat Rev Microbiol.

[18] Poretsky RS, Bano N, Buchan A, LeCleir G, Kleikemper J, Pickering M, Pate WM, Moran MA, Hollibaugh JT (2005). Analysis of microbial gene transcripts in environmental samples. Appl Environ Microbiol.

[19] Gilbert JA, Field D, Huang Y, Edwards R, Li W, Gilna P, Joint I (2008). Detection of large numbers of novel sequences in the metatranscriptomes of complex marine microbial communities. PloS One.

[20] Keeling PJ, Burki F, Wilcox HM, Allam B, Allen EE, Amaral-Zettler LA, Armbrust EV, Archibald JM, Bharti AK, Bell CJ (2014). The marine microbial eukaryote transcriptome sequencing project (MMETSP): illuminating the functional diversity of eukaryotic life in the oceans through transcriptome sequencing. PLoS Biol.

[21] Krinos AI, Hu SK, Cohen NR, Alexander H (2021). EUKulele: taxonomic annotation of the unsung eukaryotic microbes. J Open Source Softw.

[22] Carradec Q, Pelletier E, Da Silva C, Alberti A, Seeleuthner Y, Blanc-Mathieu R, Lima-Mendez G, Rocha F, Tirichine L, Labadie K (2018). A global ocean atlas of eukaryotic genes. Nat Commun.

[23] Alexander H, Jenkins BD, Rynearson TA, Dyhrman ST (2015). Metatranscriptome analyses indicate resource partitioning between diatoms in the field. Proc Natl Acad Sci.

[24] Johnson LK, Alexander H, Brown CT (2019). Re-assembly, quality evaluation, and annotation of 678 microbial eukaryotic reference transcriptomes. Gigascience.

[25] Daniels C, Baumgarten S, Yum LK, Michell CT, Bayer T, Arif C, Roder C, Weil E, Voolstra CR (2015). Metatranscriptome analysis of the reef-building coral *Orbicella faveolata* indicates holobiont response to coral disease. Front Mar Sci.

[26] Lesniewski RA, Jain S, Anantharaman K, Schloss PD, Dick GJ (2012). The metatranscriptome of a deep-sea hydrothermal plume is dominated by water column methanotrophs and lithotrophs. ISME J.

[27] Richter D (2017). Metagenomics and metatranscriptomes of oceanic communities. Phycologia.

[28] Leimena MM, Ramiro-Garcia J, Davids M, van den Bogert B, Smidt H, Smid EJ, Boekhorst J, Zoetendal EG, Schaap PJ, Kleerebezem M (2013). A comprehensive metatranscriptome analysis pipeline and its validation using human small intestine microbiota datasets. BMC Genomics.

[29] Davids M, Hugenholtz F, dos Santos VM, Smidt H, Kleerebezem M, Schaap PJ (2016). Functional profiling of unfamiliar microbial communities using a validated de novo assembly metatranscriptome pipeline. PloS One.

[30] Westreich ST, Treiber ML, Mills DA, Korf I, Lemay DG (2018). SAMSA2: a standalone metatranscriptome analysis pipeline. BMC Bioinform.

[31] Vijay N, Poelstra JW, Künstner A, Wolf JB (2013). Challenges and strategies in transcriptome assembly and differential gene expression quantification. A comprehensive in silico assessment of RNA-seq experiments. Mol Ecol.

[32] MacManes MD (2018). The Oyster River Protocol: a multi-assembler and kmer approach for de novo transcriptome assembly. PeerJ.

[33] Ortiz R, Gera P, Rivera C, Santos JC (2021). Pincho: a modular approach to high quality de novo transcriptomics. Genes.

[34] Simão FA, Waterhouse RM, Ioannidis P, Kriventseva EV, Zdobnov EM (2015). BUSCO: assessing genome assembly and annotation completeness with single-copy orthologs. Bioinformatics.

[35] Jauhal AA, Newcomb RD. Assessing genome assembly quality prior to downstream analysis: N50 versus BUSCO. Molecular Ecology Resources. 2021. 10.1111/1755-0998.13364 33629477

[36] Bushmanova E, Antipov D, Lapidus A, Prjibelski AD (2019). rnaSPAdes: a de novo transcriptome assembler and its application to RNA-Seq data. GigaScience.

[37] Jiang Y, Xiong X, Danska J, Parkinson J (2016). Metatranscriptomic analysis of diverse microbial communities reveals core metabolic pathways and microbiome-specific functionality. Microbiome.

[38] Almeida A, Mitchell AL, Tarkowska A, Finn RD (2018). Benchmarking taxonomic assignments based on 16S rRNA gene profiling of the microbiota from commonly sampled environments. GigaScience.

[39] Anwar MZ, Lanzen A, Bang-Andreasen T, Jacobsen CS (2019). To assemble or not to resemble-a validated comparative metatranscriptomics workflow (CoMW). GigaScience.

[40] Bolger AM, Lohse M, Usadel B (2014). Trimmomatic: a flexible trimmer for Illumina sequence data. Bioinformatics.

[41] Bushnell B. BBMap: a fast, accurate, splice-aware aligner. Technical report, Lawrence Berkeley National Lab. (LBNL), Berkeley, CA (United States). 2014.

[42] Honaas LA, Wafula EK, Wickett NJ, Der JP, Zhang Y, Edger PP, Altman NS, Pires JC, Leebens-Mack JH, DePamphilis CW (2016). Selecting superior de novo transcriptome assemblies: lessons learned by leveraging the best plant genome. PLoS One.

[43] Clarke K, Yang Y, Marsh R, Xie L (2013). Comparative analysis of de novo transcriptome assembly. Sci China Life Sci.

[44] Namiki T, Hachiya T, Tanaka H, Sakakibara Y (2012). MetaVelvet: an extension of Velvet assembler to de novo metagenome assembly from short sequence reads. Nucleic Acids Res.

[45] Simpson JT, Durbin R (2012). Efficient de novo assembly of large genomes using compressed data structures. Genome Res.

[46] Grabherr MG, Haas BJ, Yassour M, Levin JZ, Thompson DA, Amit I, Adiconis X, Fan L, Raychowdhury R, Zeng Q (2011). Full-length transcriptome assembly from RNA-Seq data without a reference genome. Nat Biotechnol.

[47] Li D, Liu C-M, Luo R, Sadakane K, Lam T-W (2015). MEGAHIT: an ultra-fast single-node solution for large and complex metagenomics assembly via succinct de Bruijn graph. Bioinformatics.

[48] Nurk S, Meleshko D, Korobeynikov A, Pevzner PA (2017). metaSPAdes: a new versatile metagenomic assembler. Genome Res.

[49] Cerveau N, Jackson DJ (2016). Combining independent de novo assemblies optimizes the coding transcriptome for nonconventional model eukaryotic organisms. BMC Bioinform.

[50] Mirdita M, Steinegger M, Söding J (2019). MMseqs2 desktop and local web server app for fast, interactive sequence searches. Bioinformatics.

[51] El-Gebali S, Mistry J, Bateman A, Eddy SR, Luciani A, Potter SC, Qureshi M, Richardson LJ, Salazar GA, Smart A (2019). The Pfam protein families database in 2019. Nucleic Acids Res.

[52] Haas B, Papanicolaou A. TransDecoder identifies candidate coding regions within transcript sequences. 2021.

[53] Huerta-Cepas J, Forslund K, Coelho LP, Szklarczyk D, Jensen LJ, Von Mering C, Bork P (2017). Fast genome-wide functional annotation through orthology assignment by eggNOG-mapper. Mol Biol Evol.

[54] Kanehisa M, et al., The KEGG database. In: Novartis Foundation Symposium, Wiley Online Library; 2002. pp. 91–100. 12539951

[55] Shannon CE (1948). A mathematical theory of communication. Bell Syst Tech J.

[56] Brown CT, Irber L (2016). sourmash: a library for MinHash sketching of DNA. J Open Source Softw.

[57] Jain C, Rodriguez-R LM, Phillippy AM, Konstantinidis KT, Aluru S (2018). High throughput ANI analysis of 90K prokaryotic genomes reveals clear species boundaries. Nat Commun.

[58] Emms DM, Kelly S (2019). OrthoFinder: phylogenetic orthology inference for comparative genomics. Genome Biol.

[59] Emms DM, Kelly S (2015). OrthoFinder: solving fundamental biases in whole genome comparisons dramatically improves orthogroup inference accuracy. Genome Biol.

[60] Liao Y, Smyth GK, Shi W (2019). The R package Rsubread is easier, faster, cheaper and better for alignment and quantification of RNA sequencing reads. Nucleic Acids Res.

[61] Haas BJ, Papanicolaou A, Yassour M, Grabherr M, Blood PD, Bowden J, Couger MB, Eccles D, Li B, Lieber M (2013). De novo transcript sequence reconstruction from RNA-seq using the Trinity platform for reference generation and analysis. Nat Protoc.

[62] Hölzer M, Marz M (2019). De novo transcriptome assembly: a comprehensive cross-species comparison of short-read RNA-Seq assemblers. Gigascience.

[63] Bushmanova E, Antipov D, Lapidus A, Suvorov V, Prjibelski AD (2016). rnaQUAST: a quality assessment tool for de novo transcriptome assemblies. Bioinformatics.

[64] Steinegger M, Söding J (2017). MMseqs2 enables sensitive protein sequence searching for the analysis of massive data sets. Nat Biotechnol.

[65] Steinegger M, Söding J (2018). Clustering huge protein sequence sets in linear time. Nat Commun.

[66] Klemetsen T, Raknes IA, Fu J, Agafonov A, Balasundaram SV, Tartari G, Robertsen E, Willassen NP (2018). The MAR databases: development and implementation of databases specific for marine metagenomics. Nucleic Acids Res.

[67] Virtanen P, Gommers R, Oliphant TE, Haberland M, Reddy T, Cournapeau D, Burovski E, Peterson P, Weckesser W, Bright J (2020). SciPy 1.0: fundamental algorithms for scientific computing in Python. Nat Methods.

[68] R Core Team: R: A Language and Environment for Statistical Computing. R Foundation for Statistical Computing, Vienna, Austria. R Foundation for Statistical Computing. 2021. https://www.R-project.org/

[69] Vorobev A, Dupouy M, Carradec Q, Delmont TO, Annamalé A, Wincker P, Pelletier E (2020). Transcriptome reconstruction and functional analysis of eukaryotic marine plankton communities via high-throughput metagenomics and metatranscriptomics. Genome Res.

[70] Sunagawa S, Coelho LP, Chaffron S, Kultima JR, Labadie K, Salazar G, Djahanschiri B, Zeller G, Mende DR, Alberti A (2015). Structure and function of the global ocean microbiome. Science.

[71] Patro R, Duggal G, Love MI, Irizarry RA, Kingsford C (2017). Salmon provides fast and bias-aware quantification of transcript expression. Nat Methods.

[72] Zerbino DR, Birney E (2008). Velvet: algorithms for de novo short read assembly using de Bruijn graphs. Genome Res.

[73] Altschul SF, Gish W, Miller W, Myers EW, Lipman DJ (1990). Basic local alignment search tool. J Mol Biol.

[74] Van Rossum G, Drake FL Jr. Python Reference Manual. Centrum voor Wiskunde en Informatica Amsterdam; 1995.

[75] Kibirige H, Lamp G, Katins J, gdowding, austin, matthias-k, Funnell T, Finkernagel F, Arnfred J, Blanchard D, Astanin S, Chiang E, Kishimoto PN, Sheehan E, stonebig, Willers, B, Gibboni R, smutch, Halchenko, Y, Pavel, King, B, RK M, Collins J, zachcp, Anthony, Koopman, B, Grohmann CH, Becker D, Brown D, Saiz D. Has2k1/plotnine: V0.8.0. 10.5281/zenodo.4636791.

[76] Wickham H. Ggplot2: elegant graphics for data analysis. Springer; 2016. (https://ggplot2.tidyverse.org).

[77] Pedersen TL. patchwork: the composer of plots. R package version 1.1.1. 2020. https://CRAN.R-project.org/package=patchwork

[78] Altschul SF, Madden TL, Schäffer AA, Zhang J, Zhang Z, Miller W, Lipman DJ (1997). Gapped BLAST and PSI-BLAST: a new generation of protein database search programs. Nucleic Acids Res.

[79] Camacho C, Coulouris G, Avagyan V, Ma N, Papadopoulos J, Bealer K, Madden TL (2009). BLAST+: architecture and applications. BMC Bioinform.

[80] Budak H, Kaya SB, Cagirici HB (2020). Long non-coding RNA in plants in the era of reference sequences. Front Plant Sci.

[81] Rogato A, Richard H, Sarazin A, Voss B, Navarro SC, Champeimont R, Navarro L, Carbone A, Hess WR, Falciatore A (2014). The diversity of small non-coding RNAs in the diatom *Phaeodactylum tricornutum*. BMC Genom.

[82] Lopez-Gomollon S, Beckers M, Rathjen T, Moxon S, Maumus F, Mohorianu I, Moulton V, Dalmay T, Mock T (2014). Global discovery and characterization of small non-coding RNAs in marine microalgae. BMC Genom.

[83] Canesi KL, Rynearson TA (2016). Temporal variation of* Skeletonema* community composition from a long-term time series in Narragansett Bay identified using high-throughput DNA sequencing. Mar Ecol Prog Ser.

[84] Damon C, Lehembre F, Oger-Desfeux C, Luis P, Ranger J, Fraissinet-Tachet L, Marmeisse R (2012). Metatranscriptomics reveals the diversity of genes expressed by eukaryotes in forest soils. PLoS One.

[85] Reiter T, Brooks PT, Irber L, Joslin SE, Reid CM, Scott C, Brown CT, Pierce-Ward NT (2021). Streamlining data-intensive biology with workflow systems. GigaScience.

[86] Cohen N, Alexander H, Krinos A, Hu SK, Lampe RH. Marine microeukaryote metatranscriptomics: sample processing and bioinformatic workflow recommendations for ecological applications. Front Marine Sci. 2022;858.

[87] Gilbert JA, Meyer F, Schriml L, Joint IR, Mühling M, Field D (2010). Metagenomes and metatranscriptomes from the L4 long-term coastal monitoring station in the Western English Channel. Stand Genom Sci.

[88] Nowinski B, Smith CB, Thomas CM, Esson K, Marin R, Preston CM, Birch JM, Scholin CA, Huntemann M, Clum A (2019). Microbial metagenomes and metatranscriptomes during a coastal phytoplankton bloom. Scientific Data.

[89] Vislova A, Aylward F, Sosa O, DeLong E (2016). Metatranscriptome sequence analysis reveals diel periodicity of microbial community gene expression in the ocean’s interior. Am Geophys Union.

[90] Ollison GA, Hu SK, Mesrop LY, DeLong EF, Caron DA (2021). Come rain or shine: depth not season shapes the active protistan community at station ALOHA in the North Pacific Subtropical Gyre. Deep Sea Res Part I.

[91] Hu SK, Liu Z, Alexander H, Campbell V, Connell PE, Dyhrman ST, Heidelberg KB, Caron DA (2018). Shifting metabolic priorities among key protistan taxa within and below the euphotic zone. Environ Microbiol.

[92] Vanni C, Schechter MS, Delmont TO, Eren AM, Steinegger M, Glöckner FO, Fernandez-Guerra A. AGNOSTOS-DB: a resource to unlock the uncharted regions of the coding sequence space. bioRxiv. 2021.

[93] Wagner GP, Kin K, Lynch VJ (2012). Measurement of mRNA abundance using RNA-seq data: RPKM measure is inconsistent among samples. Theory Biosci.

[94] Shakya M, Lo C-C, Chain PS. Advances and challenges in metatranscriptomic analysis. Front Genet. 2019;904. 10.3389/fgene.2019.00904 PMC6774269 31608125

[95] Vanni C, Schechter MS, Acinas SG, Barberán A, Buttigieg PL, Casamayor EO, Delmont TO, Duarte CM, Eren AM, Finn RD (2022). Unifying the known and unknown microbial coding sequence space. Elife.

[96] Berube P, Gifford S, Hurwitz B, Jenkins B, Marchetti A, Santoro A. Roadmap towards community-wide intercalibration and standardization of ocean nucleic acids ’omics measurements. 10.1575/1912/28054. https://hdl.handle.net/1912/28054

