# Correction: Reverse engineering environmental metatranscriptomes clarifies best practices for eukaryotic assembly

**DOI:** 10.1186/s12859-023-05313-0

**Published:** 2023-05-05

**Authors:** Arianna I. Krinos, Natalie R. Cohen, Michael J. Follows, Harriet Alexander

**Affiliations:** 1 MIT-WHOI Joint Program in Oceanography and Applied Ocean Science and Engineering, Cambridge and Woods Hole, MA USA; 2 Department of Biology, Woods Hole Oceanographic Institution, Woods Hole, MA USA; 3 Department of Earth, Atmospheric, and Planetary Science, Massachusetts Institute of Technology, Cambridge, MA USA; 4 Skidaway Institute of Oceanography, University of Georgia, Savannah, GA USA

**Correction to: Krinos et al. BMC Bioinformatics (2023) 24:74** 10.1186/s12859-022-05121-y

Following the publication of the original article [1], the authors identified that legends of Figs. 8 and  9 were swapped. Below the legends are correctly related to the figures.

**Fig. 8:**
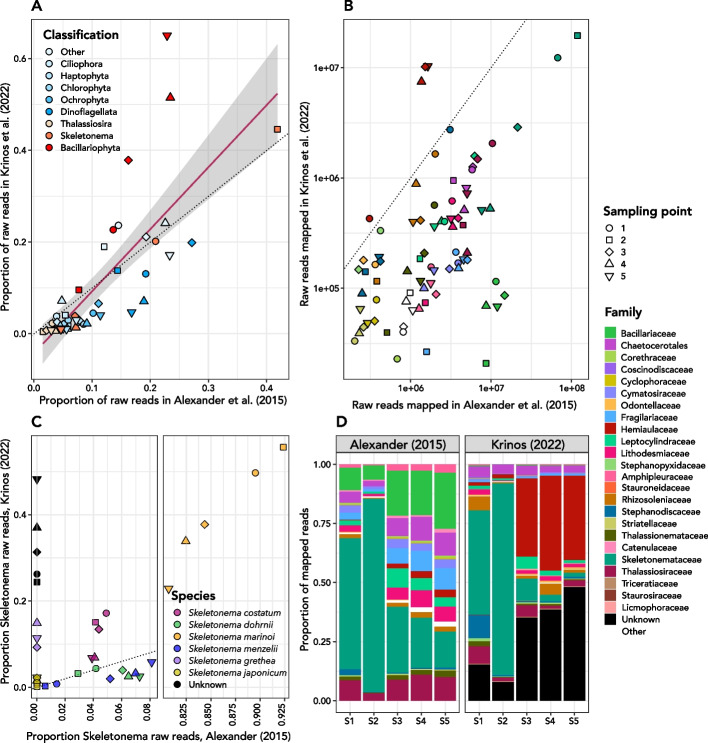
Narragansett Bay dataset from Alexander et al. (2015) [23] assembled using *euk*rhythmic. **A** The correspondence between the proportion of total raw reads in (y) this study vs. (x) [23]. Each point represents a sampling time, and *Bacillariophyta* aggregates all non-*Skeletonema* and non-*Thalassiosira* diatoms. **B** Family-level taxonomic breakdown of [23]’s raw read mapping (left) as compared to this study. **C** Log-normalized raw reads mapped to each taxonomic family compared between the two studies. **D** *Skeletonema* species represented in the *euk*rhythmic reassembly representing some of the diversity within this genus known to show seasonal dominance in Narragansett Bay

**Fig. 9:**
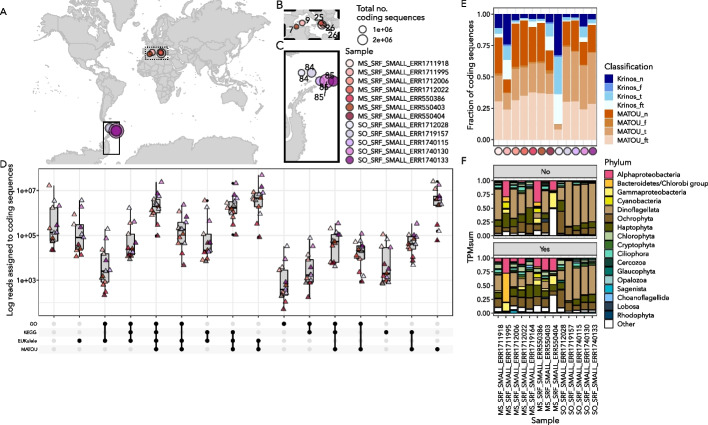
*Tara* Oceans reassemblies using *euk*rhythmic (Carradec et al. (2018) [22]). **A** Map showing the locations of reassembled *Tara* Oceans samples. Boxes over regions are expanded in Panels B and C. **B** Mediterranean Sea samples. Numbers indicate *Tara* Oceans stations. **C** Southern Ocean samples. As in Panel B, numbers indicate *Tara* Oceans stations. **D** between-assembler overlap of the reads assigned to coding sequences. The x-axis indicates the annotations assigned to each of the coding sequences, and the y-axis shows the between-sample sum of reads assigned to coding sequences for that category. **E** Fraction of coding sequences that did or did not have a match to the MATOU database. Shades of blue indicate coding sequences recovered only by this study. The top segment indicates coding sequences without functional or taxonomic annotations, following by the proportion of sequences with functional and taxonomic annotations (“ft”), the proportion with only functional annotations (“f”), and the proportion with only taxonomic annotations (“t”). The same is shown in shades of orange for the assembld coding sequences from this study that did have a significant match to the MATOU database. The y-axis shows the color-coded *Tara* Oceans sample. F: The fraction of TPM assigned to coding sequences with recovered taxonomic annotations. These are from the “Not in MATOU” “ft” and “t” bars in Panel E. Dinoflagellated dominate many of the Southern Ocean samples, particularly for those coding sequences which could not be taxonomically annotated

The original article [1] has been corrected.
