# Supplementary material for: Reverse engineering environmental metatranscriptomes clarifies best practices for eukaryotic assembly
Source: BMC Bioinformatics. 2023 Mar 3;24:74. doi: 10.1186/s12859-022-05121-y (PMC9983209; doi:10.1186/s12859-022-05121-y)
Supplement: Supplementary file 1 — Additional file 1: Fig. S1. Visual summary of the effect of clustering with mmseqs2 on recovered enera, KOs (functional annotations) and file size in bytes. While reducing the equence identity threshold for clustering results in up to a 30% average reduction in file size, he number of recovered genera and functional annotations are only modestly impacted, specially at high coverage. An intermediate sequence identity of 0.8 and coverage of .8 would result in a 15-25% average reduction in file size, but leave distinct functional and taxonomic annotations unchanged. Fig. S2. Main text figure facetted by simulated METSP assembly group (two different sets of organisms). Of note is that the eukrhythmic reassembly accurately recapitulates the bimodal distribution in GC-content observed in MMETSP group A’s designer metatranscriptomic sequences. Fig. S3. Protein sequence lengths in the reassemblies as compared to the designer. The 1-to-1 line shows where sequences would fall if the average length of recovered protein sequences via TransDecoder were identical between the designer assemblies and the eukrhythmic-derived reassembled products; the fact that all samples fall in the lower right half of the plot indicates that protein sequences were consistently larger in the designer assemblies as compared to the eukrhythmic reassembled products. Fig. S4. Mean contig length as a function of the number of assemblers that found a sequence that matched the given description. For the 4-assembler cluster, this means that all four assemblers tested identified a sequence that matched the sequence included in the distribution when clustered within eukrhythmic. Panel A corresponds to MMETSP Group A while Panel B corresponds to MMETSP Group B. Welch’s independent T-tests and Kolmogorov-Smirnoff tests for between-distribution goodness of fit computed on these length distributions reveals that the overall distribution of lengths for 1 vs. 2 vs. 3 vs. 4-assembler distributions are all statistic [file 12859_2022_5121_MOESM1_ESM.pdf]

Supplemental Figures

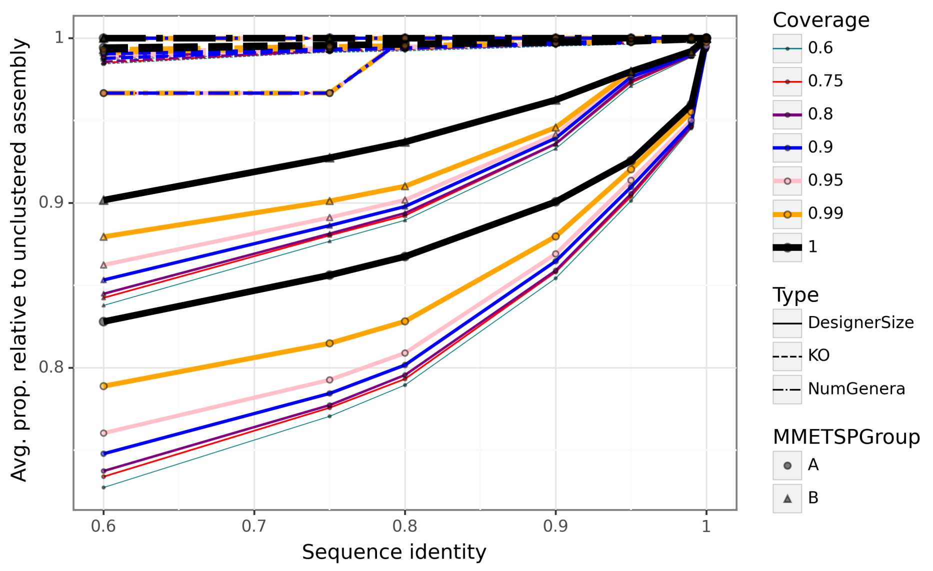

Figure 1 Visual summary of the effect of clustering with `mmseqs2` on recovered genera, KOs (functional annotations) and file size in bytes. While reducing the sequence identity threshold for clustering results in up to a 30% average reduction in file size, the number of recovered genera and functional annotations are only modestly impacted, especially at high coverage. An intermediate sequence identity of 0.8 and coverage of 0.8 would result in a 15-25% average reduction in file size, but leave distinct functional and taxonomic annotations unchanged.

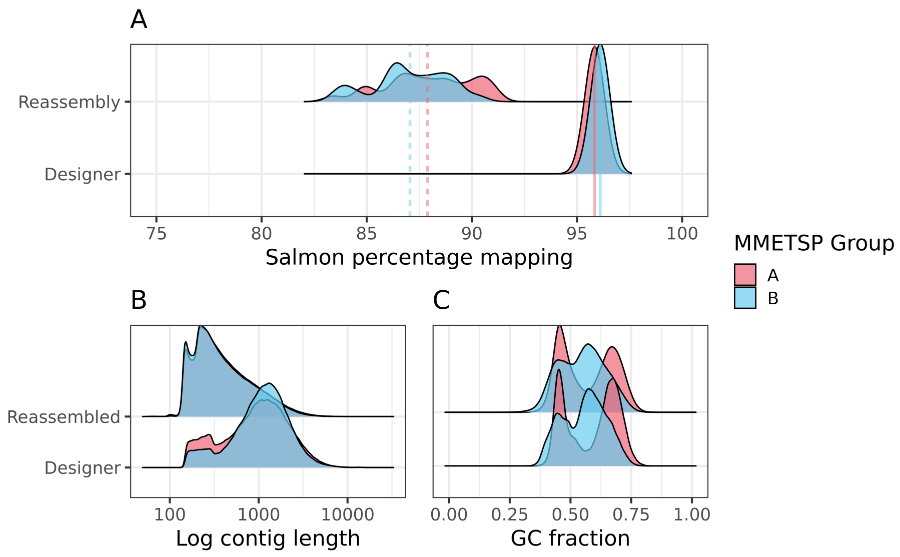

Figure 2 Main text figure facetted by simulated MMETSP assembly group (two different sets of organisms). Of note is that the eukrhythmic reassembly accurately recapitulates the bimodal distribution in GC-content observed in MMETSP group A's designer metatranscriptomic sequences.

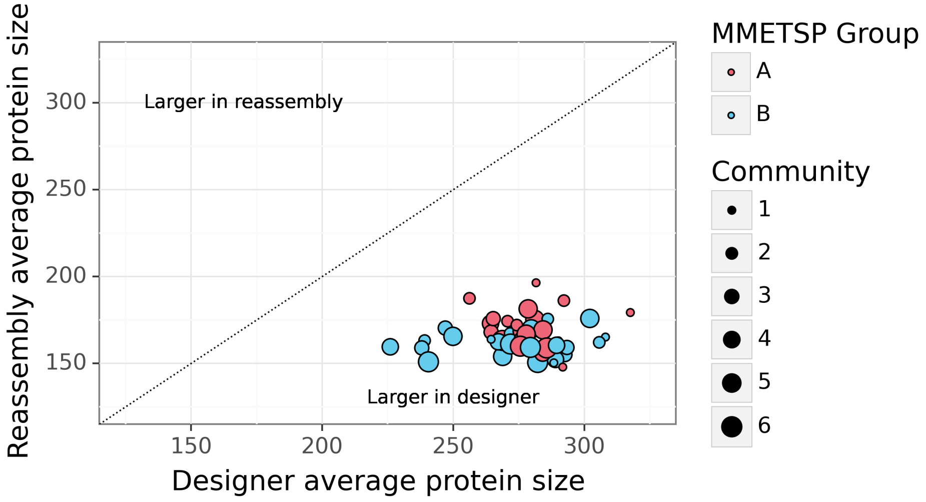

Figure 3 Protein sequence lengths in the reassemblies as compared to the designer. The 1-to-1 line shows where sequences would fall if the average length of recovered protein sequences via `TransDecoder` were identical between the designer assemblies and the eukrhythmic-derived reassembled products; the fact that all samples fall in the lower right half of the plot indicates that protein sequences were consistently larger in the designer assemblies as compared to the eukrhythmic reassembled products.

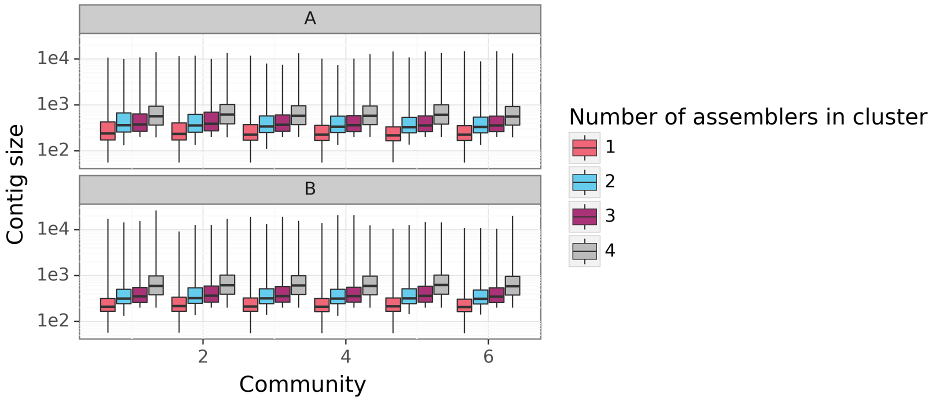

Figure 4 Mean contig length as a function of the number of assemblers that found a sequence that matched the given description. For the 4-assembler cluster, this means that all four assemblers tested identified a sequence that matched the sequence included in the distribution when clustered within eukrhythmic. Panel A corresponds to MMETSP Group A while Panel B corresponds to MMETSP Group B. Welch's independent T-tests and Kolmogorov-Smirnoff tests for between-distribution goodness of fit computed on these length distributions reveals that the overall distribution of lengths for 1 vs. 2 vs. 3 vs. 4-assembler distributions are all statistically significantly different ($p < 1e-6$), with greater assemblers within a cluster leading to higher average length.

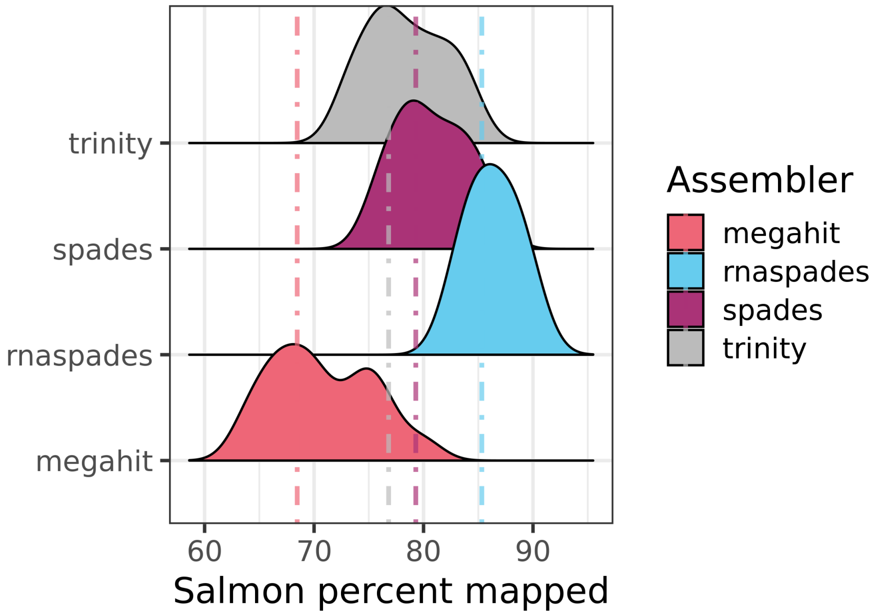

Figure 5 Salmon mapping percentages against the simulated raw reads when computed individually against each of the four assembly tools used by eukrhythmic. rnaSPAdes consistently outperformed the other assemblers with regard to percentage mapping, average length, and number of annotations.

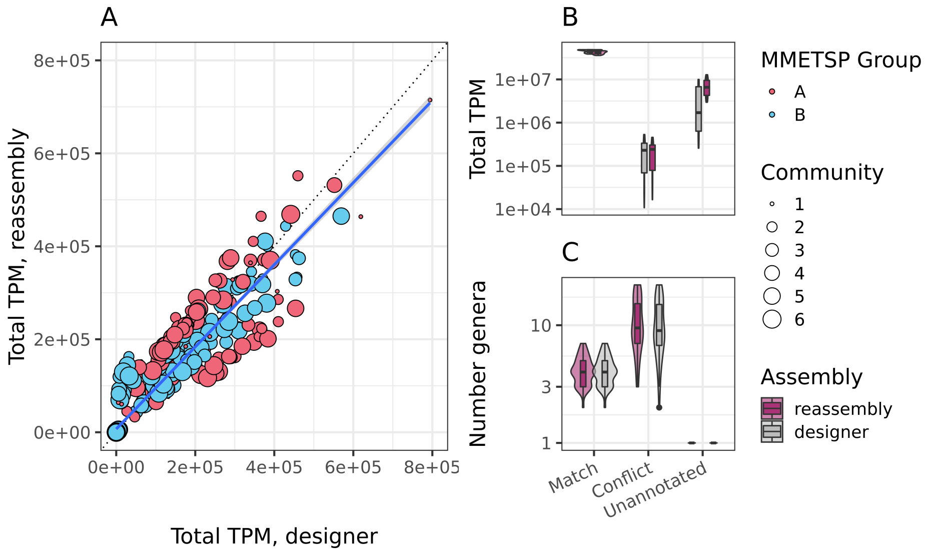

Figure 6 Main text figure, but with A: TPM comparison for the designer assemblies and the reassembled products from `eukrhythmic` labelled by distinct groupings of simulations relative to their subset of MMETSP organisms; each point is filled according to its "MMETSP group".

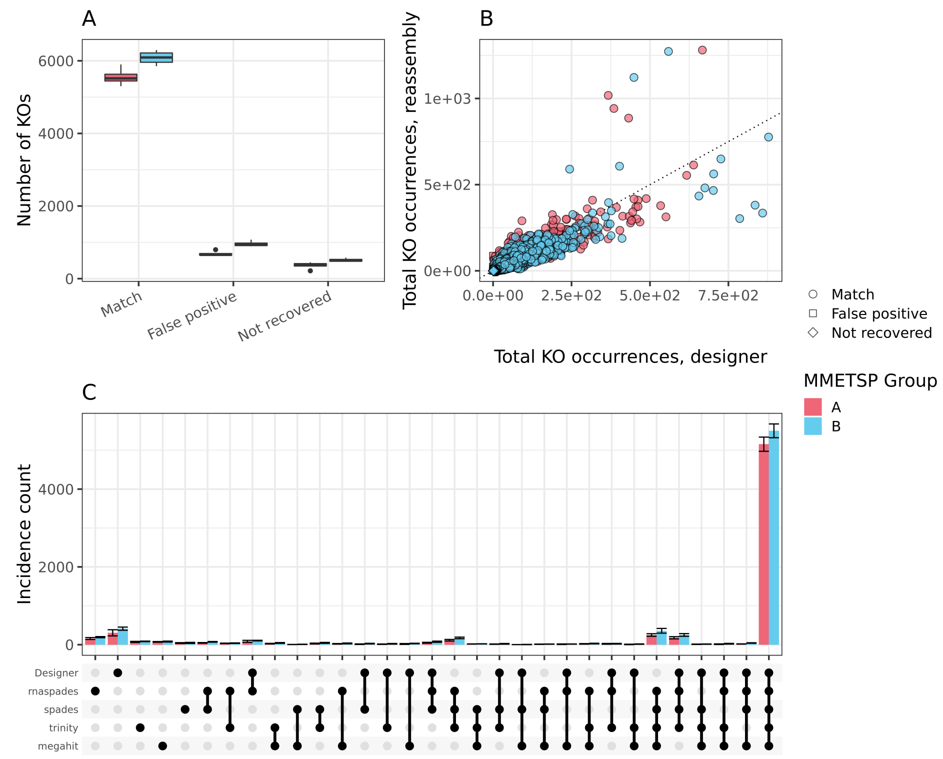

Figure 7 Main text figure with the results split by "MMETSP group" to demonstrate that different taxonomic groupings of organisms included in the simulation does not impact the general trends observed in the results. All three panels are divided by samples using the two "MMETSP groups" of individual organism transcriptomes.

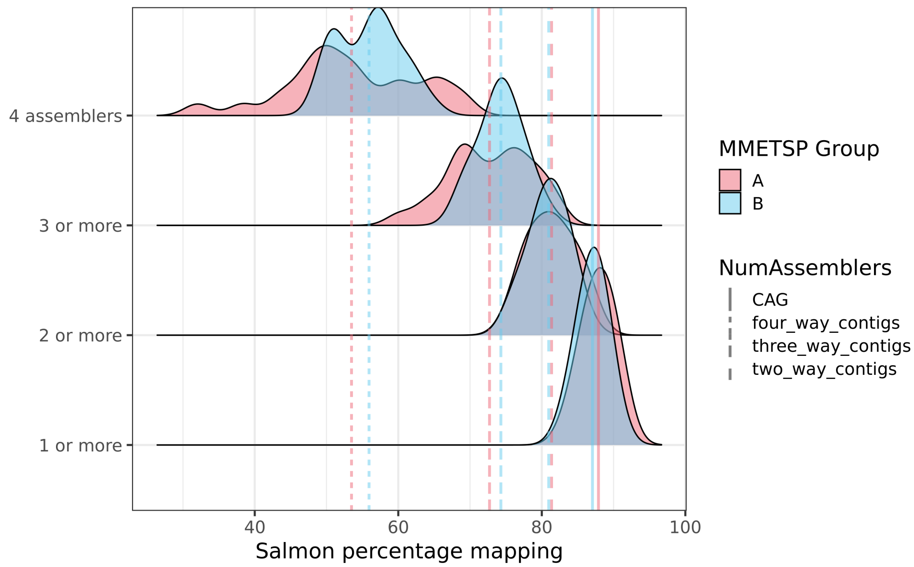

Figure 8 Salmon percentage mapping by MMETSP group when contig products from all assemblers as in eukrhythmic are used (bottom distribution with one or more assembler) as compared to when only contigs agreed upon by multiple assemblers are used. Average percentage mapping decreases progressively with fewer contigs being included as the criteria for inclusion are made stricter.

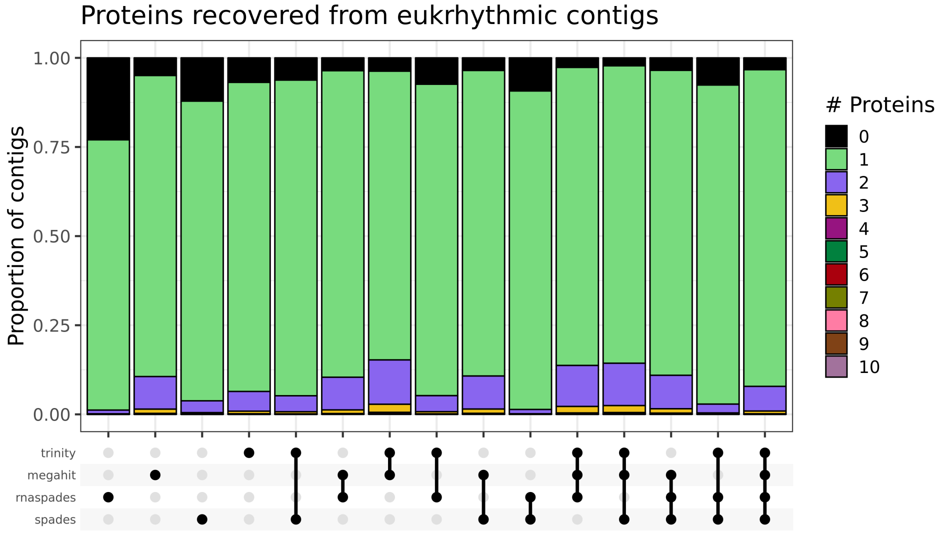

Figure 9 Proportion of contigs from each clustering subset that had ORFs extracted from the sequence. The vast majority of contigs had a single predicted ORF, but `rnaSPAdes` alone had the greatest number of nucleotide contigs on which an ORF could not be detected. In practice, these sequences might be assumed to be non-coding.

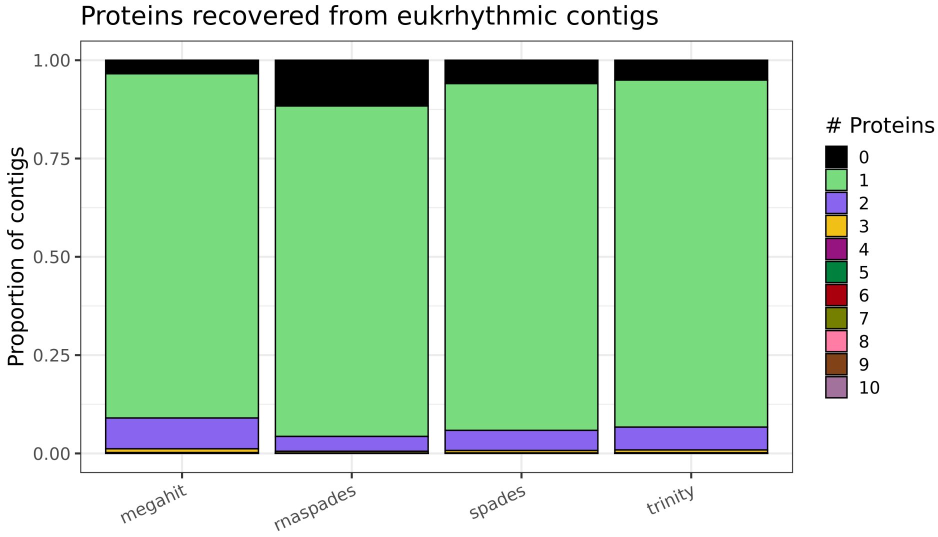

Figure 10 Proportion of contigs from each clustering subset that had ORFs extracted from the sequence across all contigs identified by the assembler. `rnaSPAdes` had a higher number of contigs without an identified ORF.

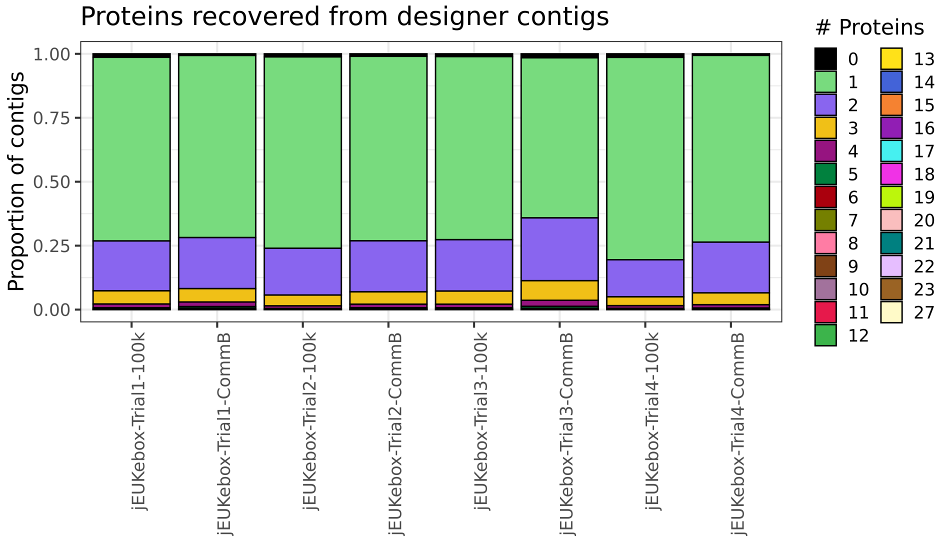

Figure 11 Proportion of contigs from the designer assembly that had ORFs extracted from the sequence. In general, fewer contigs did not have an identified ORF, and a higher number of contigs had multiple predicted ORFs than in the reassembled products from `eukrhythmic`.

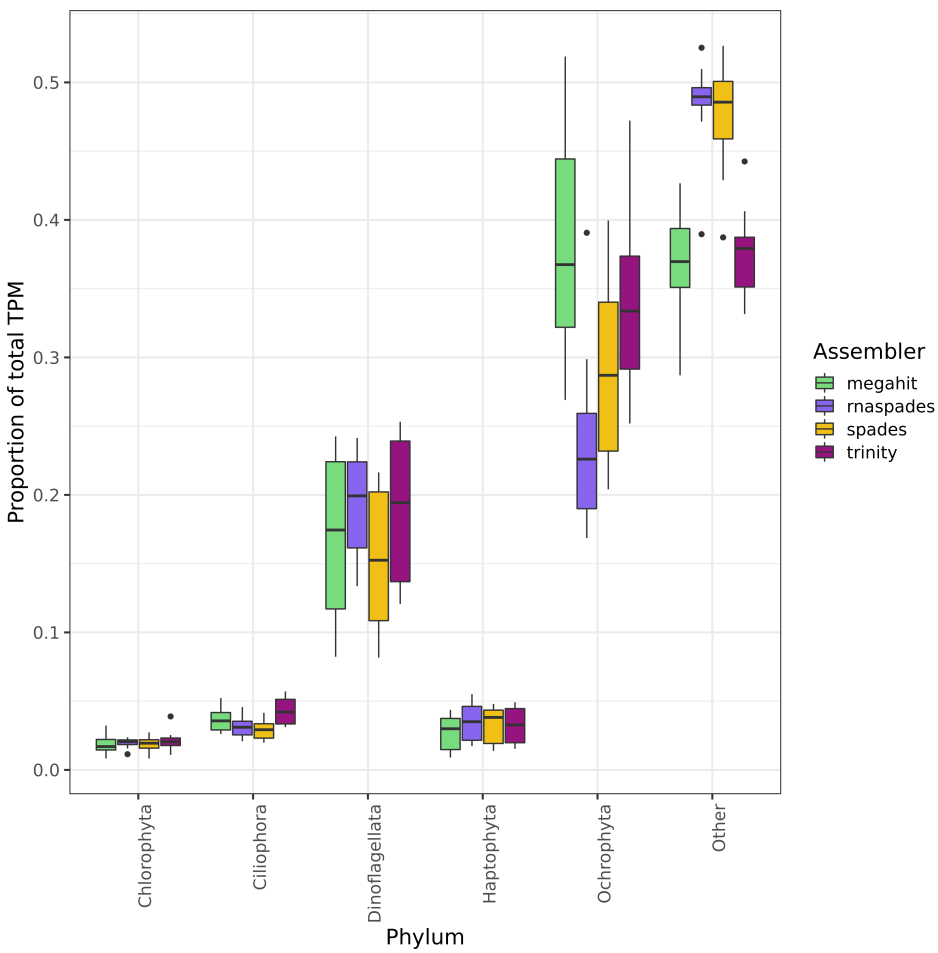

Figure 12 Comparison of the abundance of KO IDs within functional annotations across Narragansett Bay samples and different combinations of metatranscriptome assemblers. A black dotted line indicates a one-to-one relationship, meaning that the abundance of KOs that fall along this line are exactly as abundant using the assembler listed on the x-axis and using the assembler listed on the y-axis. On the top left, `Trinity` is compared to `MEGAHIT`, on the top right `Trinity` is compared to `rnaSPAdes`, on the bottom left `MEGAHIT` is compared to `rnaSPAdes` and on the bottom right `MEGAHIT` is compared to `SPAdes`. Each point corresponds to a single KO within a sample. While `rnaSPAdes` tended to report high abundances of each identified KO relative to the other assemblers, `MEGAHIT` reported fewer instances of each KO than the other three assemblers in most samples. This may be due to the approaches adopted by the two assemblers. Whereas in a typical assembly, k-mers that appear only once are assumed to be the result of error, these k-mers may represent real and important diversity in a set of low-abundance whole-community sequences [?]. The `MEGAHIT` assembler is an example of metagenomics-specific software that defines "mercy k-mers" which come into play in between two k-mers within a single read that are sequenced *more* than once. `rnaSPAdes`, as an example, does not employ a "mercy" strategy but instead decreases the coverage threshold significantly in comparison to genomic assembly [?]. One is adopting the meta-omic assumption that exceedingly low coverage is plausibly not artificial, while the other is being more generous with respect to coverage and memory use, but intending merely to minimize the influence of sequencing error. The strategy adopted by `MEGAHIT` may result in incomplete consideration of isoforms, which could have contributed to the relatively low recovery rate of multiple copies of the identified KO groups by the `MEGAHIT` assemblies of the Narragansett Bay metatranscriptome samples.

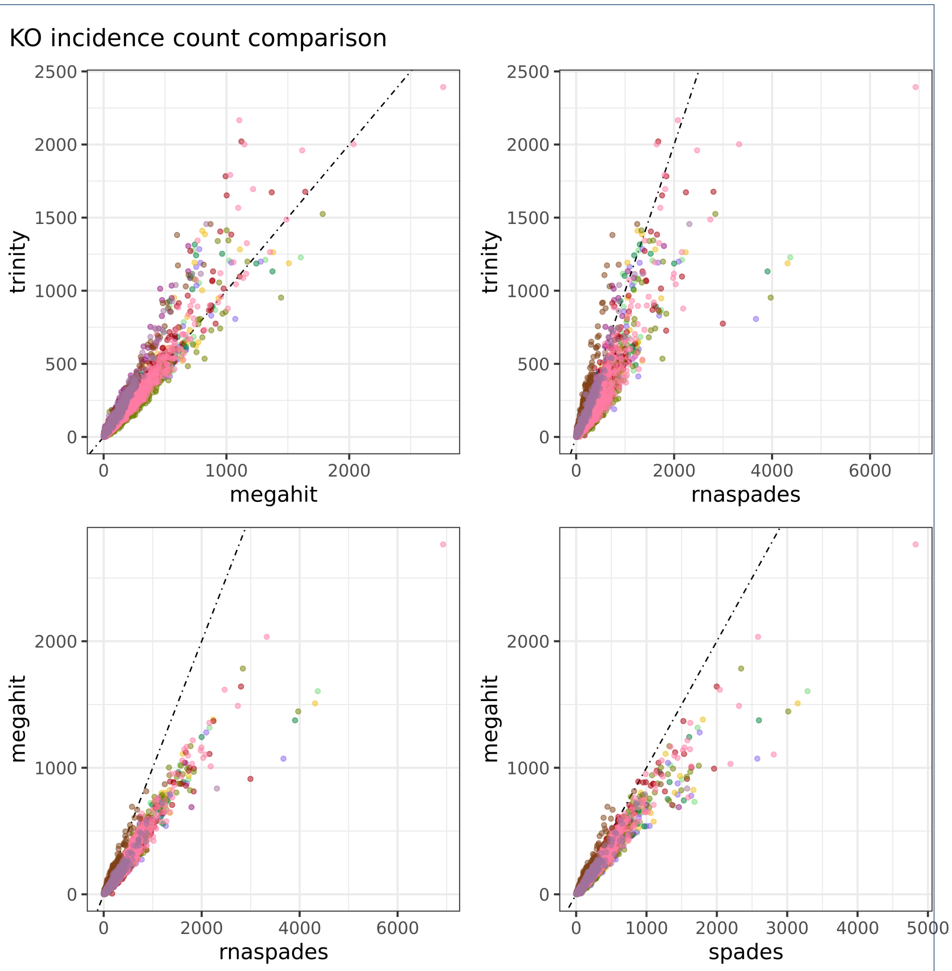

Figure 13 Proportion of total normalized TPM value of major taxonomic groups according to the four metatranscriptomic assemblers that were tested. `rnaSPAdes` had a lower proportion of TPM assigned to Ochrophyta (including diatoms), but further investigation appeared this to be in large part a consequence of the large number of small contigs produced by `rnaSPAdes`.

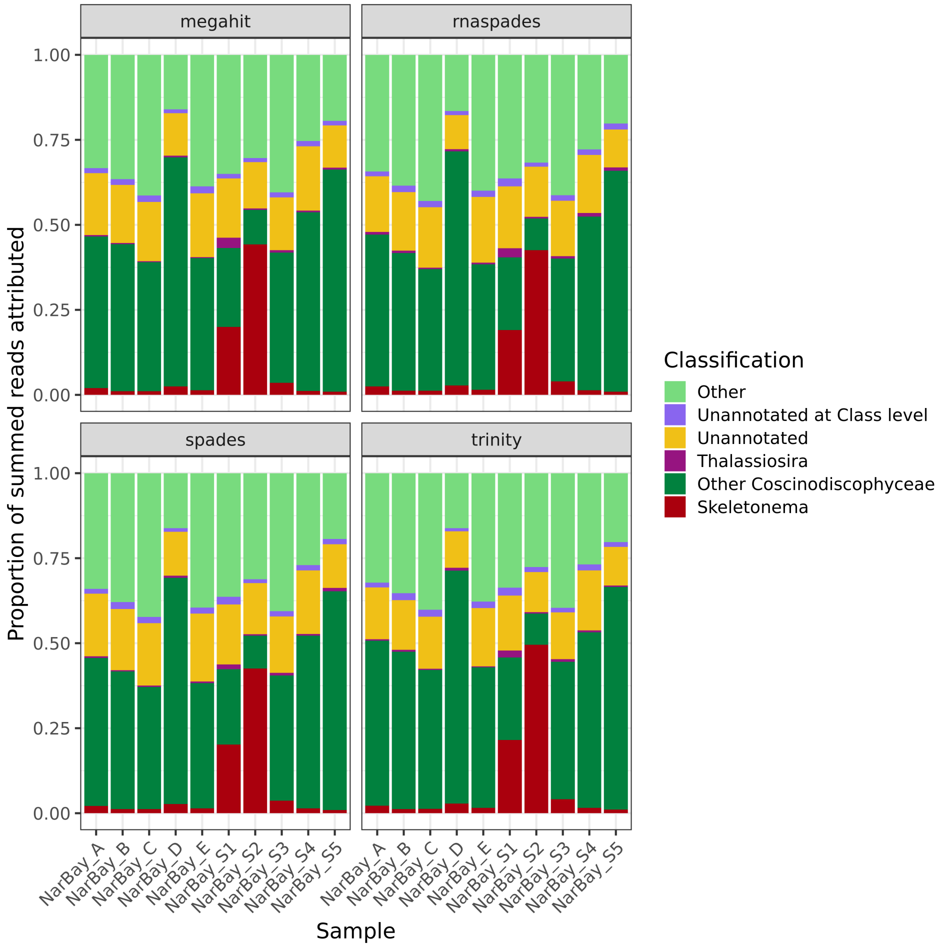

Figure 14 Proportion of total normalized TPM as reported by `Salmon` from the raw reads assigned to each taxonomic category in the assemblies produced by each of the four assemblers (facets). In particular, in sample S2 the taxonomic breakdown of the eukaryotic community differs importantly in the midst of a bloom of the diatom *Skeletonema*.

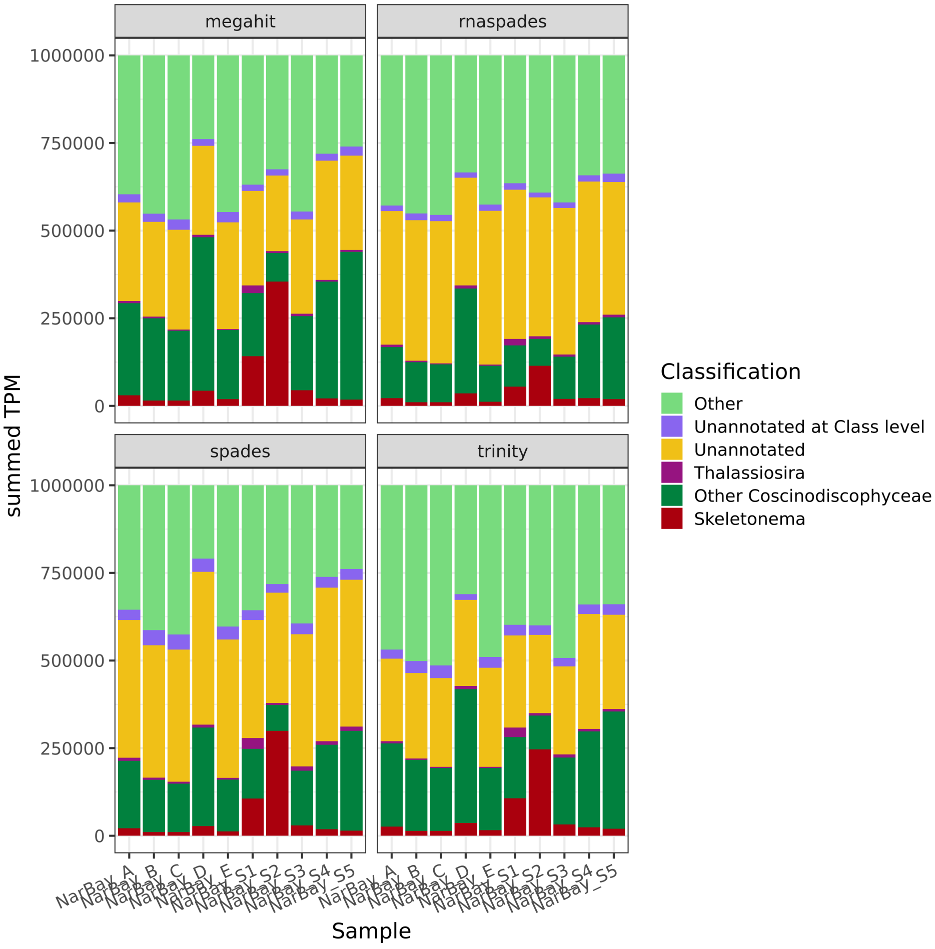

Figure 15 Proportion of assigned raw reads as reported by `Salmon` assigned to each taxonomic category for the four metatranscriptome assemblers, expressed as a proportion of the total.

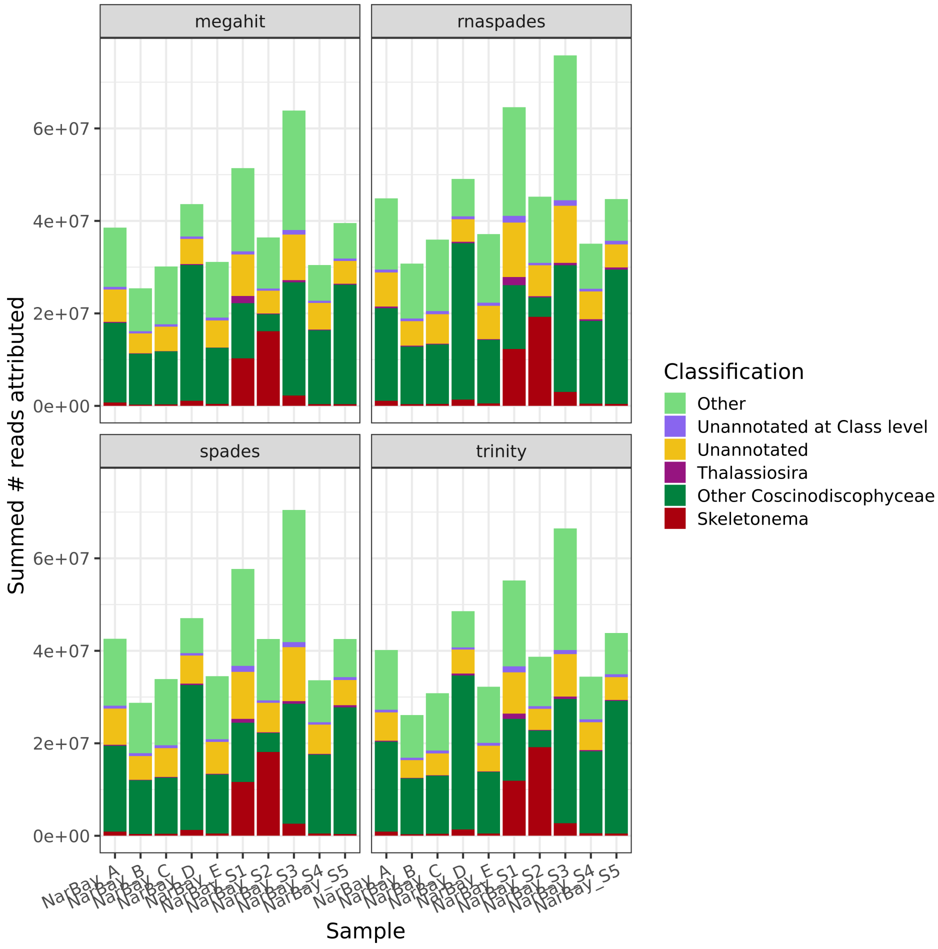

Figure 16 Proportion of assigned raw reads as reported by `Salmon` assigned to each taxonomic category for the four metatranscriptome assemblers, expressed as a proportion of the total.

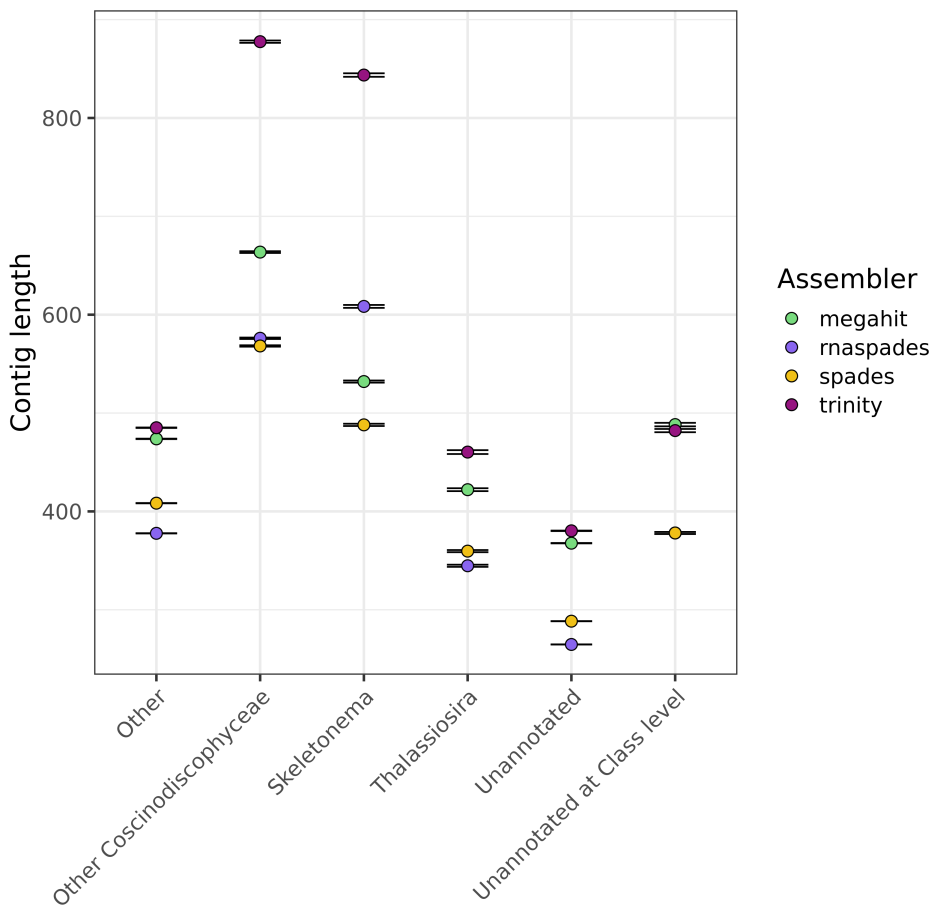

Figure 17 Average contig length by annotation of contigs generated by the four assembly tools across samples. Error bars show the standard error of the mean.

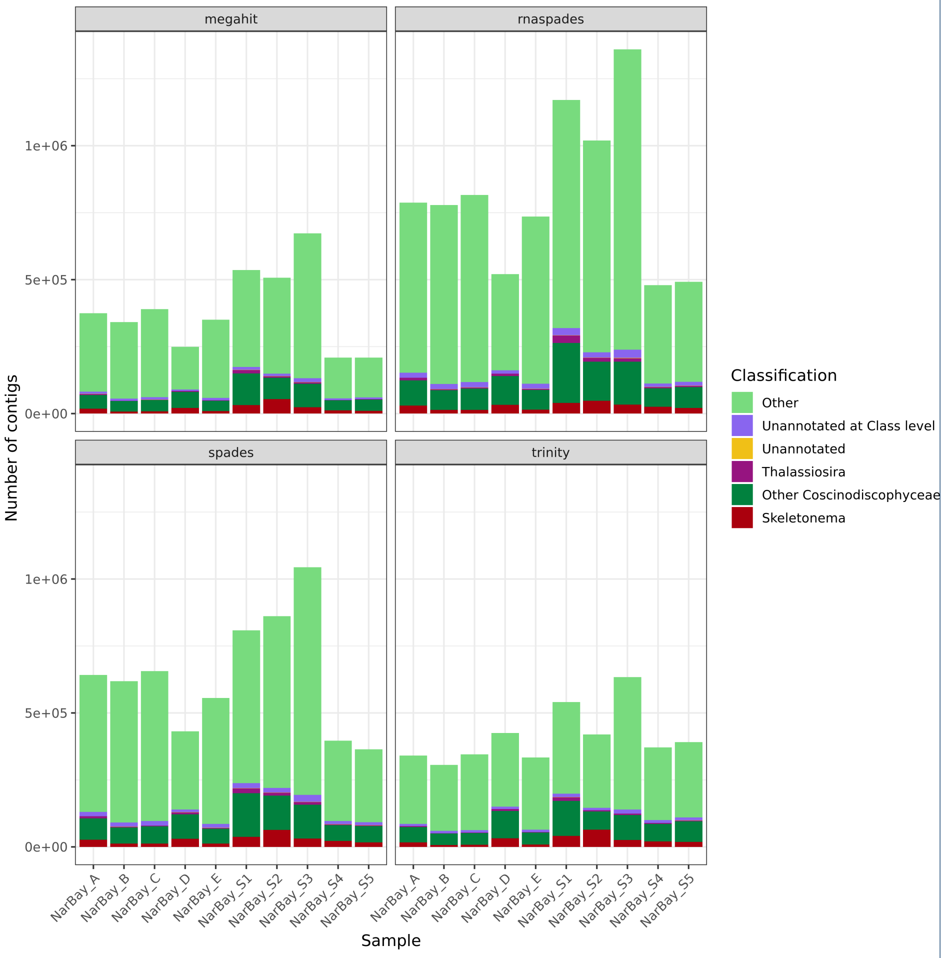

Figure 18 Total number of contigs generated by the four tested assemblers for each of the taxonomic groupings considered. `rnaSPAdes` tended to produce more contigs than the other four assemblers, but these contigs were often shorter and occasionally led to misleading community composition results.

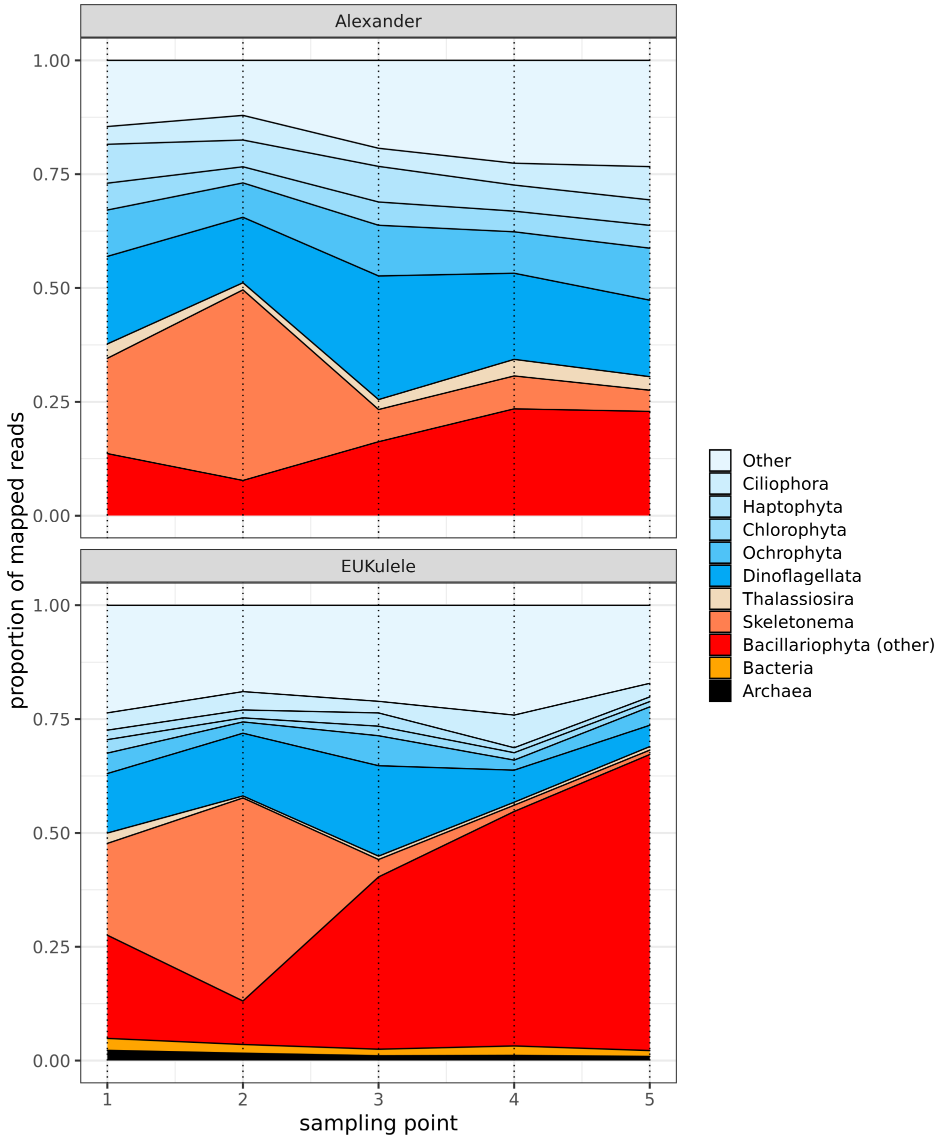

Figure 19 Analogous stacked abundance plots by taxonomic grouping to the relative abundance plots presented in [?] for the five in-situ sampling points collected from Narragansett Bay.

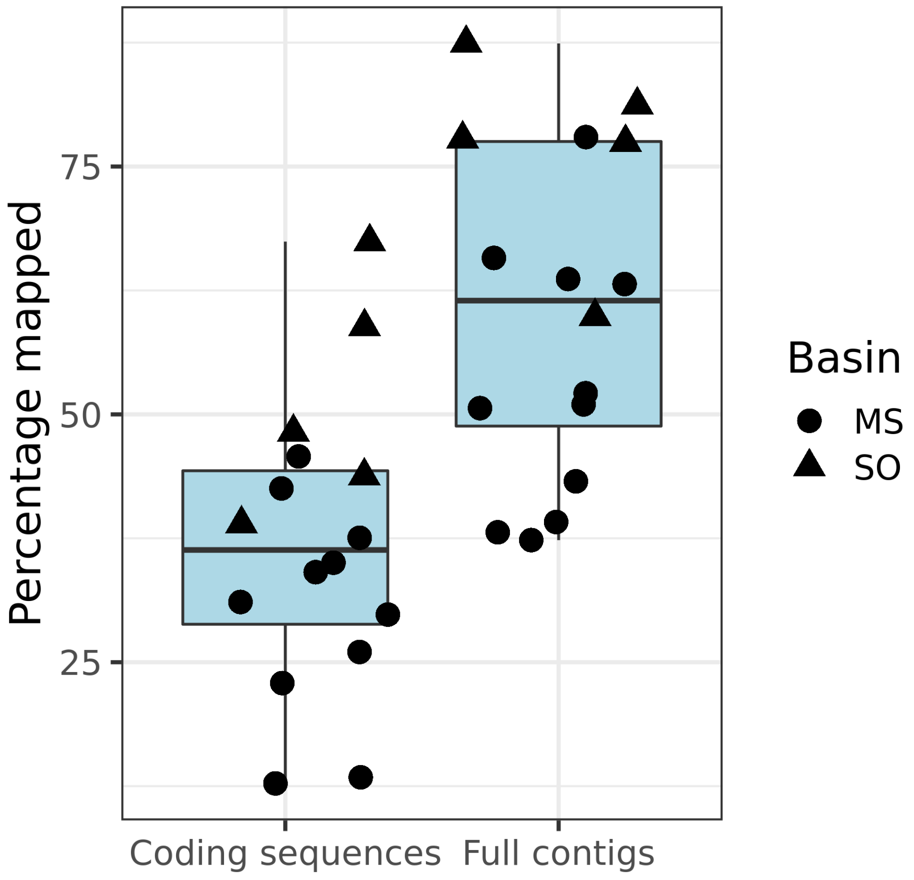

Figure 20 Salmon percentage mapping of coding sequences (left) vs. entire contigs (right) for the *Tara* Oceans samples. Mapping only to coding sequences from the assembly decreased the mean percentage mapped, as reported by Salmon.

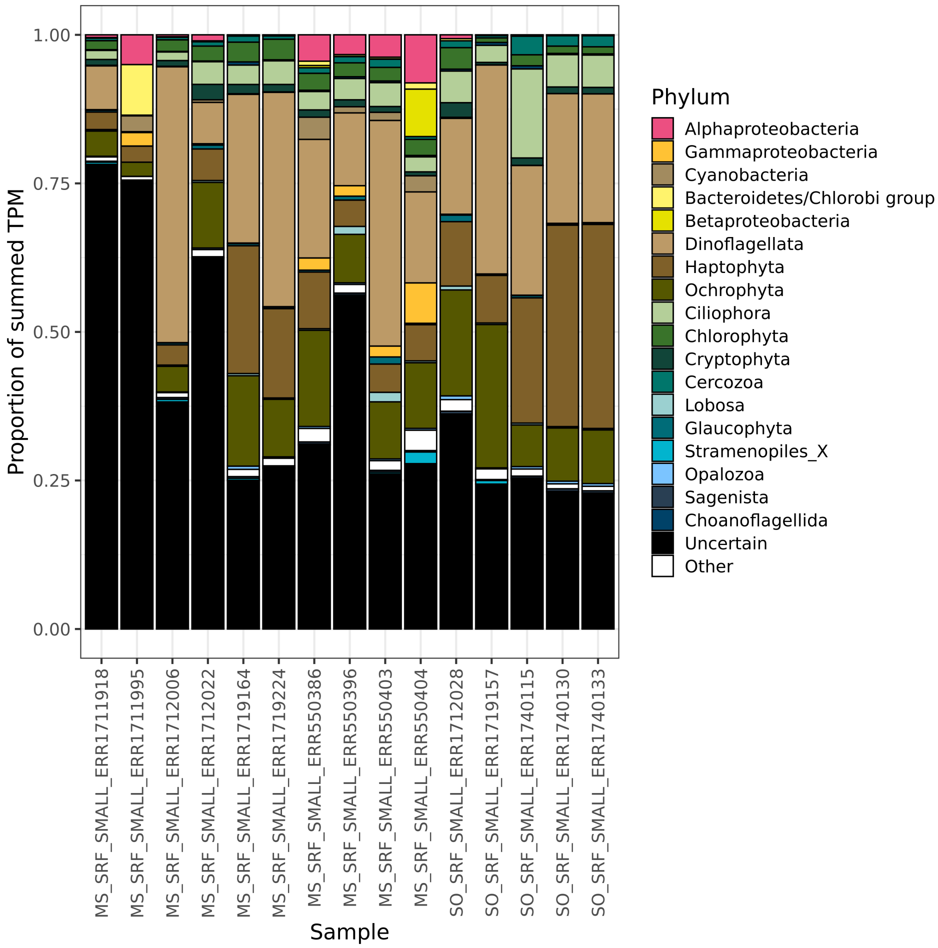

Figure 21 Per-sample taxonomic annotations for all *euk*`rhythmic` CDSs, including those which were and were not found to have a match to the MATOU database.

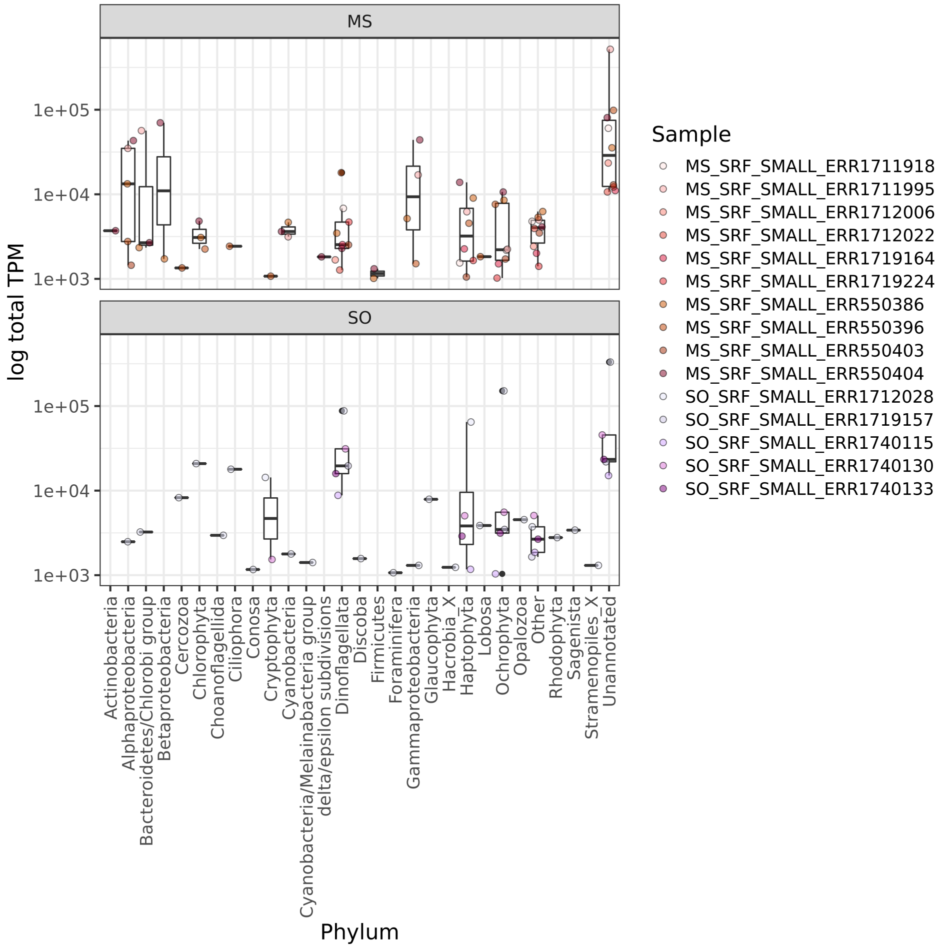

Figure 22 Total TPM assigned per sample to sequences that were assigned a `EUKulele` annotation, but did not have a significant `blast` match to the MATOU database from [?].

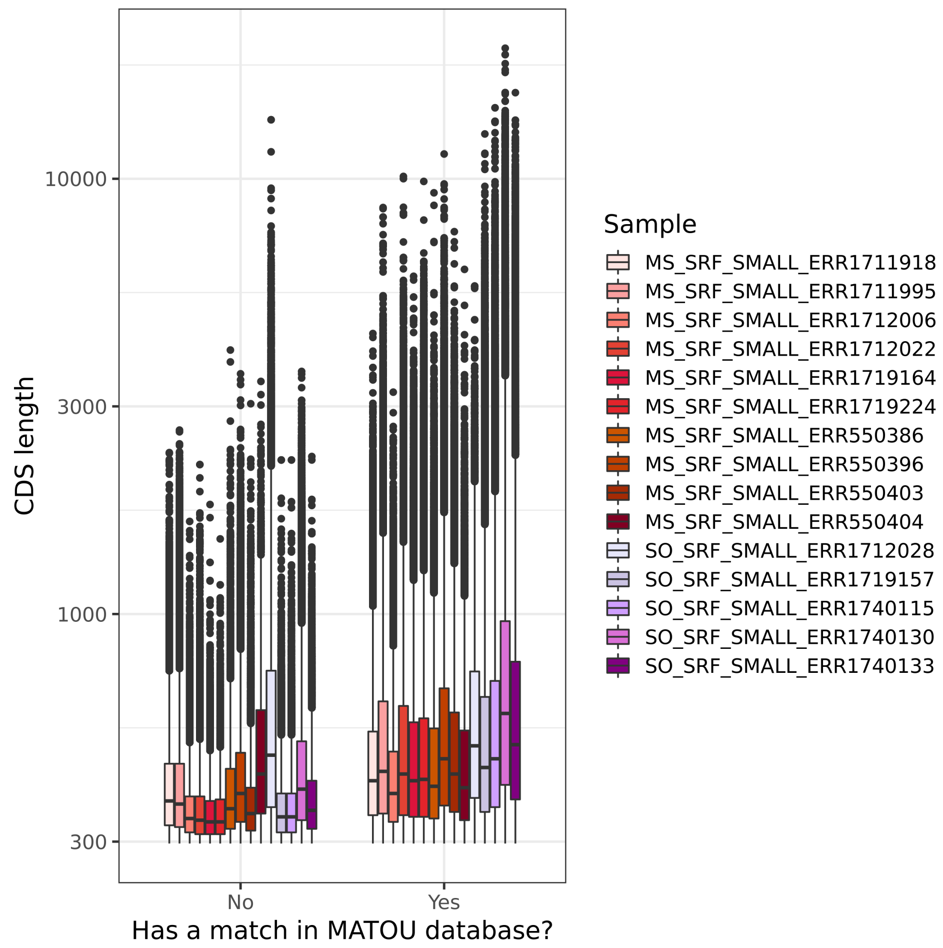

Figure 23 Full length distribution of coding sequences recovered by the eukrhythmic assembly and were (left) not found in the MATOU database [?] vs. (right) found in the MATOU database.

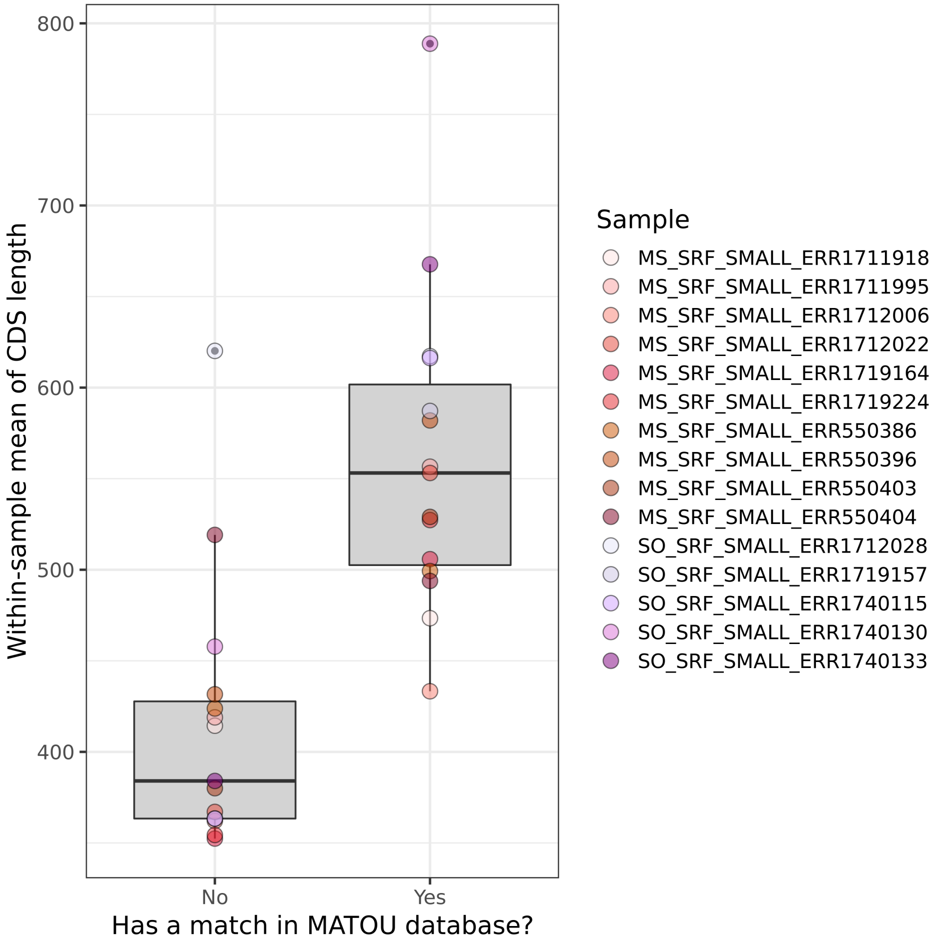

Figure 24 Per-sample average length of coding sequences recovered by the eukrhythmic assembly and were (left) not found in the MATOU database [?] vs. (right) found in the MATOU database.

Table 1 (Supplement) Effect of clustering designer assembly on assembly size and annotations. Clustering was performed on the original "designer metatranscriptome" set of contigs from the MMETSP references using the `mmseqs2` tool [?]. Eukrhythmic uses a coverage level of 0.98 and sequence identity of 1 for `mmseqs2` clustering. See Figure ?? Supplement for a graphical summary of the influence of sequence identity and coverage on the size of the recovered assembly and its functional and taxonomic annotations.

Supplementary Tables

MMETSP Group	Cluster Seq. ID	Cluster Cover-age	No. Contigs	File Size (MB)	No. Genera	No. Species	No. KOs
A	0.6	0.6	35515 ± 6240	21.2 ± 3.7	3.6 ± 0.8	4.4 ± 1.2	4803.5 ± 403.1
A	0.6	1.0	40793 ± 6312	24.1 ± 3.8	3.8 ± 0.8	4.4 ± 1.2	4838.9 ± 403.4
A	0.75	0.8	38426 ± 6734	22.6 ± 4.0	3.6 ± 0.8	4.4 ± 1.2	4831.1 ± 404.4
A	0.8	0.8	39422 ± 6824	23.2 ± 4.1	3.8 ± 0.8	4.4 ± 1.2	4835.2 ± 404.9
A	0.99	0.6	47174 ± 6616	27.5 ± 4.3	3.8 ± 0.8	4.4 ± 1.2	4860.3 ± 399.8
A	0.99	0.8	47282 ± 6604	27.6 ± 4.3	3.8 ± 0.8	4.4 ± 1.2	4861.3 ± 399.7
A	0.99	0.95	47541 ± 6543	27.7 ± 4.2	3.8 ± 0.8	4.4 ± 1.2	4862.6 ± 399.7
A	0.99	0.99	47937 ± 6470	27.8 ± 4.2	3.8 ± 0.8	4.4 ± 1.2	4863.0 ± 399.6
A	0.99	1.0	48152 ± 6414	28.0 ± 4.2	3.8 ± 0.8	4.4 ± 1.2	4863.4 ± 399.5
A	1.0	0.6	49595 ± 6100	28.9 ± 4.1	3.8 ± 0.8	4.4 ± 1.2	4865.6 ± 398.7
A	1.0	0.8	49672 ± 6094	28.9 ± 4.0	3.8 ± 0.8	4.4 ± 1.2	4865.8 ± 398.7
A	1.0	0.95	49807 ± 6069	29.0 ± 4.0	3.8 ± 0.8	4.4 ± 1.2	4866.4 ± 398.7
A	1.0	0.99	50036 ± 6037	29.1 ± 4.0	3.8 ± 0.8	4.4 ± 1.2	4866.5 ± 398.8
A	1.0	1.0	50164 ± 6009	29.1 ± 4.0	3.8 ± 0.8	4.4 ± 1.2	4866.8 ± 398.7
B	0.6	0.6	38425 ± 5911	24.0 ± 4.1	4.9 ± 1.4	4.9 ± 1.4	5038.8 ± 312.7
B	0.6	1.0	41618 ± 6334	25.8 ± 4.4	4.9 ± 1.4	4.9 ± 1.4	5083.9 ± 311.0
B	0.75	0.8	40848 ± 6271	25.2 ± 4.3	4.9 ± 1.4	4.9 ± 1.4	5080.0 ± 310.4
B	0.8	0.8	41550 ± 6349	25.6 ± 4.4	4.9 ± 1.4	4.9 ± 1.4	5086.6 ± 309.9
B	0.99	0.6	46150 ± 6852	28.3 ± 4.8	4.9 ± 1.4	4.9 ± 1.4	5116.3 ± 306.8
B	0.99	0.8	46202 ± 6858	28.3 ± 4.8	4.9 ± 1.4	4.9 ± 1.4	5116.4 ± 306.8
B	0.99	0.95	46276 ± 6865	28.4 ± 4.8	4.9 ± 1.4	4.9 ± 1.4	5116.6 ± 306.6
B	0.99	0.99	46369 ± 6876	28.4 ± 4.8	4.9 ± 1.4	4.9 ± 1.4	5117.0 ± 306.4
B	0.99	1.0	46391 ± 6879	28.4 ± 4.8	4.9 ± 1.4	4.9 ± 1.4	5117.2 ± 306.4
B	1.0	0.6	46580 ± 6875	28.6 ± 4.8	4.9 ± 1.4	4.9 ± 1.4	5118.2 ± 306.3
B	1.0	0.8	46616 ± 6879	28.6 ± 4.8	4.9 ± 1.4	4.9 ± 1.4	5118.3 ± 306.3
B	1.0	0.95	46660 ± 6882	28.6 ± 4.8	4.9 ± 1.4	4.9 ± 1.4	5118.5 ± 306.0
B	1.0	0.99	46722 ± 6889	28.6 ± 4.8	4.9 ± 1.4	4.9 ± 1.4	5118.9 ± 305.9
B	1.0	1.0	46753 ± 6890	28.6 ± 4.8	4.9 ± 1.4	4.9 ± 1.4	5118.9 ± 305.9

Table 1 (Supplement) Resulting assembly size and taxonomic, functional, and core content recovery of `jEUKebox` outputs after raw read simulation and re-assembly with *euk*`rhythmic`. Four assemblers were used in this analysis which were then clustered together using default *euk*`rhythmic` settings. The mean and standard deviation of four trials of each community and list of MMETSP IDs is presented.

Community	Simulated Contigs	Number of Assembled Contig Clusters	Assembly Size (MB)	MMETSP Genera	Designer Genera	Recovered Genera	MMETSP Species	Designer Species	Recovered Species	Designer Distinct KOs	Recovered Distinct KOs
1A	53239 ± 5160	33.1 ± 2.9	61254 ± 4277	3.5 ± 0.6	14.8 ± 7.1	32.2 ± 10.9	3.8 ± 0.5	19.5 ± 3.9	31.0 ± 6.7	3222.2 ± 345.4	1908.5 ± 214.9
2A	44797 ± 3236	25.7 ± 2.2	47465 ± 5357	3.0 ± 0.8	15.8 ± 10.4	29.0 ± 14.2	3.2 ± 0.5	17.0 ± 4.7	27.5 ± 9.3	3798.2 ± 118.7	1895.2 ± 66.0
3A	51653 ± 4847	29.1 ± 3.3	53644 ± 5989	4.0 ± 0.8	22.0 ± 6.7	32.8 ± 7.3	4.2 ± 1.0	21.2 ± 4.0	32.0 ± 5.0	3596.8 ± 316.6	1958.5 ± 191.0
4A	51051 ± 6771	29.2 ± 5.0	57838 ± 8582	4.0 ± 0.8	21.5 ± 8.9	36.5 ± 12.8	5.2 ± 1.0	23.2 ± 5.1	33.8 ± 9.2	3415.0 ± 198.9	1961.8 ± 188.7
5A	44538 ± 5114	25.5 ± 2.6	49321 ± 5258	3.2 ± 0.5	14.5 ± 8.4	28.5 ± 10.8	3.5 ± 0.6	18.0 ± 5.1	28.2 ± 10.7	3627.2 ± 120.2	1756.5 ± 172.6
6A	55707 ± 2467	32.1 ± 1.5	61751 ± 2621	4.8 ± 0.5	21.0 ± 7.6	37.0 ± 5.4	6.2 ± 0.5	23.5 ± 4.5	35.0 ± 2.2	3330.2 ± 109.0	1968.8 ± 175.2
1B	50243 ± 7228	32.1 ± 4.6	57724 ± 5265	4.0 ± 0.0	21.0 ± 3.7	47.8 ± 5.4	4.0 ± 0.0	15.2 ± 1.5	29.8 ± 6.0	3648.0 ± 436.4	1974.0 ± 108.7
2B	43564 ± 4108	26.6 ± 3.2	51242 ± 4351	3.5 ± 1.0	17.0 ± 4.8	38.5 ± 11.0	3.5 ± 1.0	12.0 ± 3.9	20.5 ± 6.9	3046.8 ± 75.3	1738.5 ± 249.8
3B	44071 ± 8863	26.8 ± 6.9	53115 ± 8702	4.8 ± 1.0	21.0 ± 5.2	44.0 ± 11.7	4.8 ± 1.0	12.5 ± 1.3	25.0 ± 5.5	3096.0 ± 224.9	1800.0 ± 51.0
4B	48771 ± 7071	29.8 ± 5.1	56685 ± 5643	6.2 ± 0.5	21.8 ± 3.1	54.8 ± 11.4	6.2 ± 0.5	15.0 ± 1.6	31.2 ± 1.5	3154.5 ± 111.7	1829.8 ± 68.5
5B	43985 ± 8056	26.3 ± 4.2	50763 ± 6235	4.2 ± 1.0	17.2 ± 4.3	36.8 ± 14.5	4.2 ± 1.0	11.2 ± 3.3	21.2 ± 5.5	2824.8 ± 500.9	1684.8 ± 70.1
6B	49884 ± 5766	30.3 ± 4.0	57900 ± 4692	6.5 ± 1.3	24.2 ± 7.1	54.0 ± 9.8	6.5 ± 1.3	17.8 ± 4.6	31.0 ± 7.6	2769.5 ± 271.3	1796.5 ± 83.4

More information: https://github.com/AlexanderLabWHOI/jEUKebox
